# Supplementary figures and images for: Differential contribution of two organelles of endosymbiotic origin to iron-sulfur cluster synthesis and overall fitness in Toxoplasma
Source: PLoS Pathog. 2021 Nov 18;17(11):e1010096. doi: 10.1371/journal.ppat.1010096 (PMC8639094; doi:10.1371/journal.ppat.1010096)

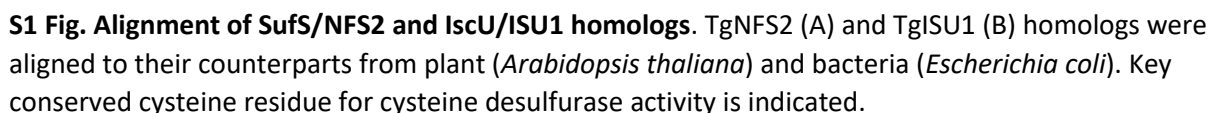

Supplement: S1 Fig — TgNFS2 (A) and TgISU1 (B) homologs were aligned to their counterparts from plant (Arabidopsis thaliana) and bacteria (Escherichia coli). Key conserved cysteine residue for cysteine desulfurase activity is indicated. (PDF) [file ppat.1010096.s001.pdf]
