# Supplementary material for: Differential contribution of two organelles of endosymbiotic origin to iron-sulfur cluster synthesis and overall fitness in Toxoplasma
Source: PLoS Pathog. 2021 Nov 18;17(11):e1010096. doi: 10.1371/journal.ppat.1010096 (PMC8639094; doi:10.1371/journal.ppat.1010096)
Supplement: S2 Fig — A) Schematic representation of the strategy for expressing HA-tagged versions of TgNFS2 (left) and TgISU1 (right) by homologous recombination at the native locus of the corresponding gene of interest. Chloramphenicol was used to select transgenic parasites based on their expression of the Chloramphenicol acetyltransferase (CAT). B) Diagnostic PCR for verifying correct integration of the construct. The amplified fragments correspond to the blue or red arrows in A), and specific primers used were ML3982/ML1476 (TgNFS2) and ML4208/ML1476 (TgISU1). (PDF) [file ppat.1010096.s002.pdf]

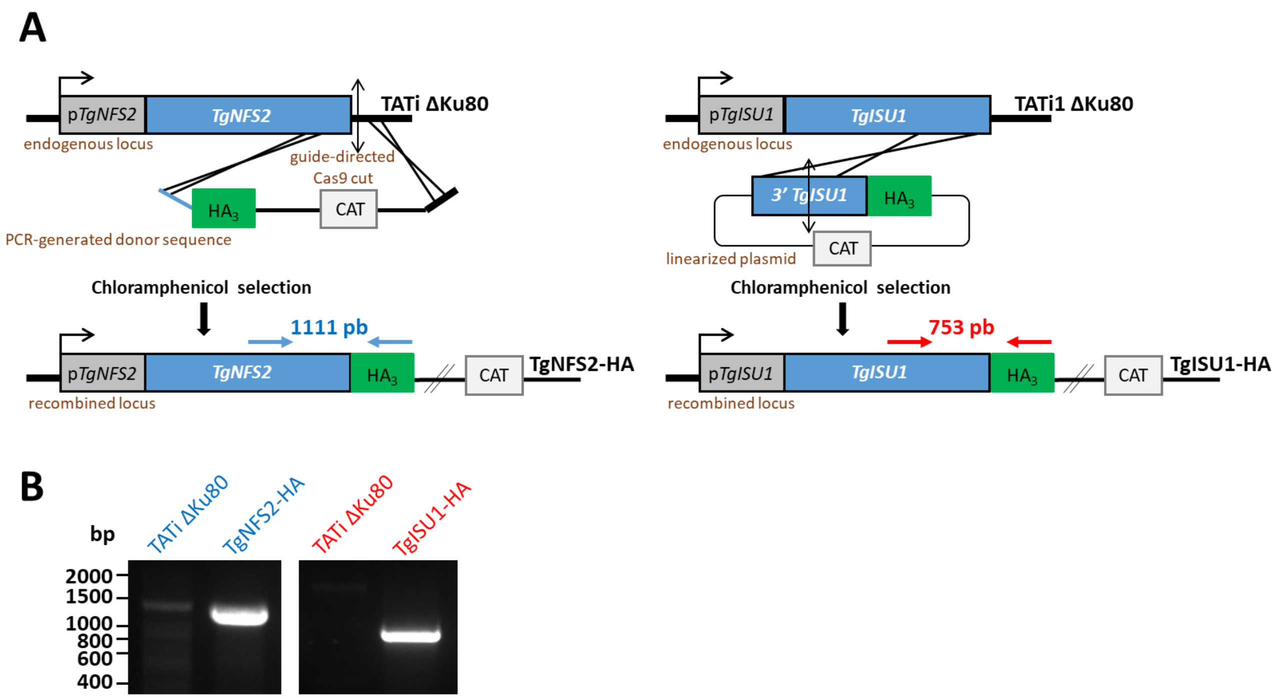

S2 Fig. Generation of HA-tagged TgNFS2 and TgISU1 cell lines. A) Schematic representation of the strategy for expressing HA-tagged versions of TgNFS2 (left) and TgISU1 (right) by homologous recombination at the native locus of the corresponding gene of interest. Chloramphenicol was used to select transgenic parasites based on their expression of the Chloramphenicol acetyltransferase (CAT). B) Diagnostic PCR for verifying correct integration of the construct. The amplified fragments correspond to the blue or red arrows in A), and specific primers used were ML3982/ML1476 (TgNFS2) and ML4208/ML1476 (TgISU1).
