# Supplementary material for: Differential contribution of two organelles of endosymbiotic origin to iron-sulfur cluster synthesis and overall fitness in Toxoplasma
Source: PLoS Pathog. 2021 Nov 18;17(11):e1010096. doi: 10.1371/journal.ppat.1010096 (PMC8639094; doi:10.1371/journal.ppat.1010096)
Supplement: S3 Fig — A) Sequence alignment of TgSUFE2 (TGGT1_277010) with plant (A. thaliana) and bacterial (E. coli) homologues. B) Schematic representation of the strategy for expressing an HA-tagged version of TgSUFE2 by double homologous recombination at the native locus. Chloramphenicol was used to select transgenic parasites based on their expression of the Chloramphenicol acetyltransferase (CAT). C) Diagnostic PCR for verifying correct integration of the construct. The amplified fragment corresponds to the red arrows in B), and specific primers used were ML4101/ML1476. D) Detection by immunoblot of C-terminally HA-tagged TgSUFE2 in parasite extracts reveals the presence of both precusor and mature forms of the protein. Anti-actin antibody (TgACT1) was used as a loading control. E) Immunofluorescence assay shows TgSUFE2 co-localizes with apicoplast marker TgCPN60. Scale bar represents 5 μm. DNA was labelled with DAPI. DIC: differential interference contrast. (PDF) [file ppat.1010096.s003.pdf]

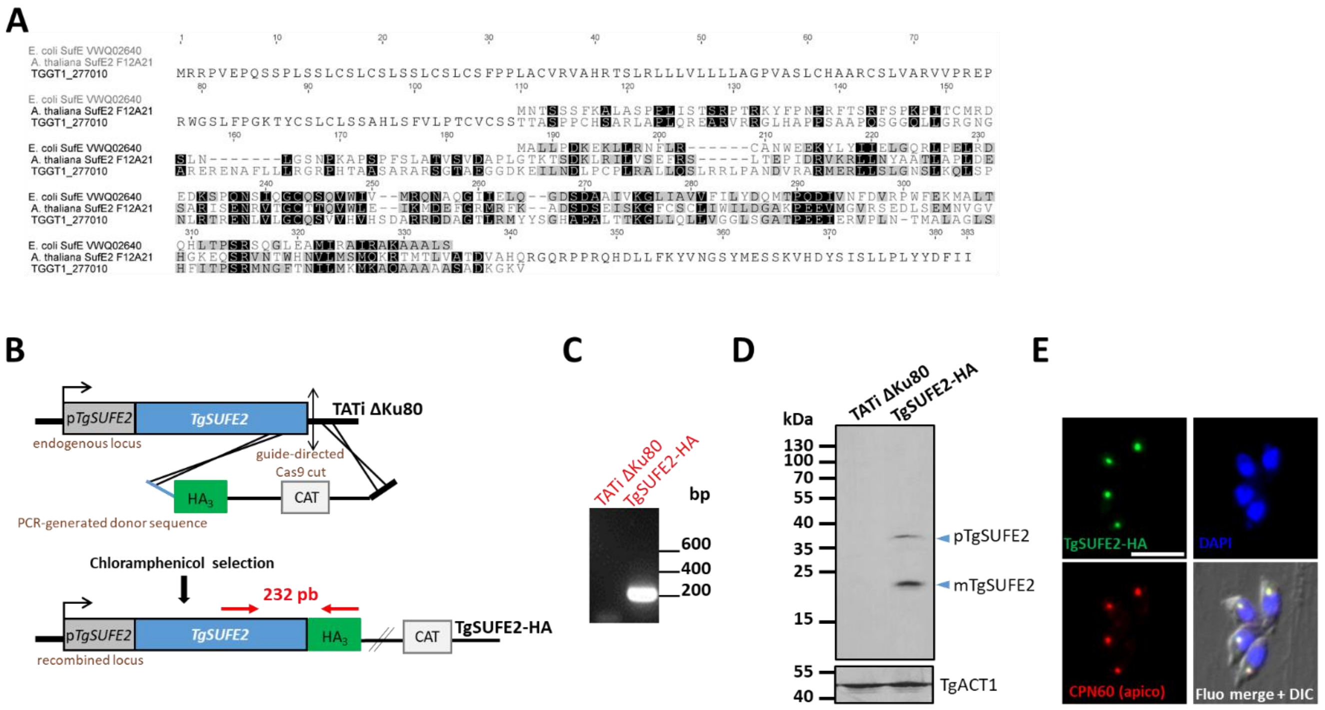

Figure S3. HA-tagging of TgSUFE2 shows it is an apicoplast protein. A) Sequence alignment of TgSUFE2 (TGGT1_277010) with plant (*A. thaliana*) and bacterial (*E. coli*) homologues. B) Schematic representation of the strategy for expressing an HA-tagged version of TgSUFE2 by double homologous recombination at the native locus. Chloramphenicol was used to select transgenic parasites based on their expression of the Chloramphenicol acetyltransferase (CAT). C) Diagnostic PCR for verifying correct integration of the construct. The amplified fragment corresponds to the red arrows in B), and specific primers used were ML4101/ML1476. D) Detection by immunoblot of C-terminally HA-tagged TgSUFE2 in parasite extracts reveals the presence of both precusor and mature forms of the protein. Anti-actin antibody (TgACT1) was used as a loading control. E) Immunofluorescence assay shows TgSUFE2 co-localizes with apicoplast marker TgCPN60. Scale bar represents 5 µm. DNA was labelled with DAPI. DIC: differential interference contrast.
