# Supplementary material for: Differential contribution of two organelles of endosymbiotic origin to iron-sulfur cluster synthesis and overall fitness in Toxoplasma
Source: PLoS Pathog. 2021 Nov 18;17(11):e1010096. doi: 10.1371/journal.ppat.1010096 (PMC8639094; doi:10.1371/journal.ppat.1010096)
Supplement: S4 Fig — A) Schematic representation of the strategy for generating TgNFS2 (top) and TgISU1 (bottom) conditional knock-down cell lines by homologous recombination at the native locus. Pyrimethamine was used to select transgenic parasites based on their expression of Dihydrofolate reductase (DHFR). B) Diagnostic PCR for verifying correct integration of the construct. The amplified fragments confirming 5’ and 3’ integration correspond to the blue and red arrows displayed in A), respectively, and specific primers used were: ML4158/ML687 (TgNFS2 5’ integration), ML1041/ML4159 (TgNFS2 3’ integration), ML1774/ML4388 (TgISU1 5’ integration), ML1771/ML4387 (TgISU1 3’ integration). (PDF) [file ppat.1010096.s004.pdf]

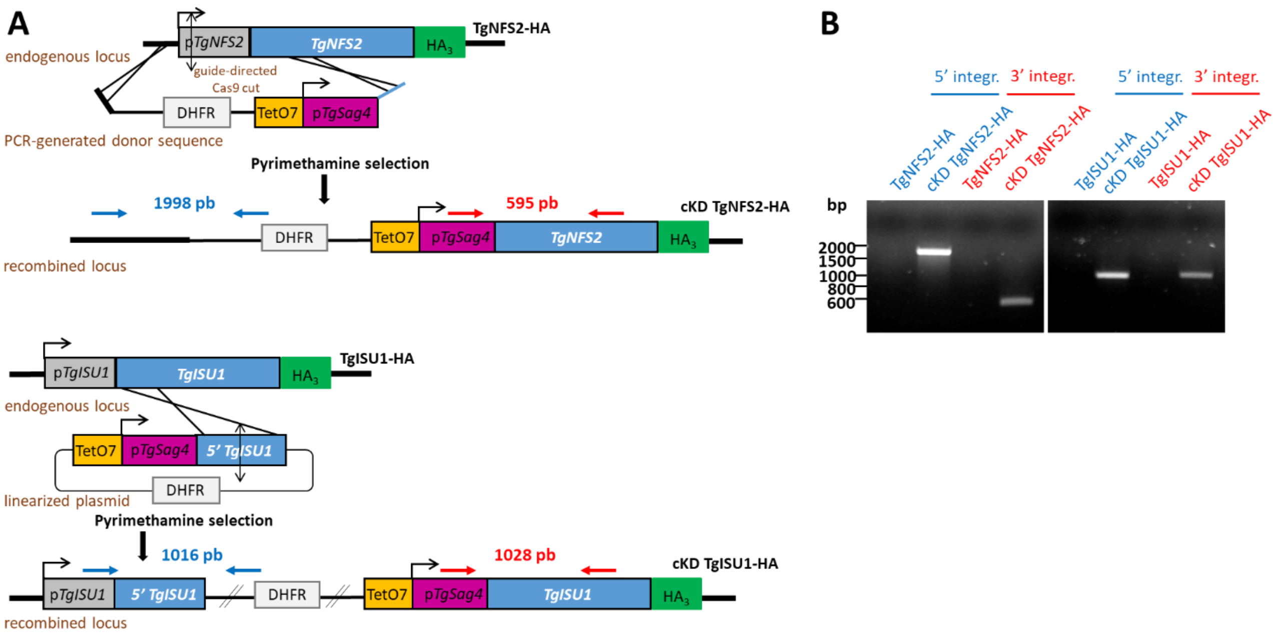

S4 Fig. Generation of TgNFS2 and TgISU1 conditional mutants. A) Schematic representation of the strategy for generating TgNFS2 (top) and TgISU1 (bottom) conditional knock-down cell lines by homologous recombination at the native locus. Pyrimethamine was used to select transgenic parasites based on their expression of Dihydrofolate reductase (DHFR). B) Diagnostic PCR for verifying correct integration of the construct. The amplified fragments confirming 5' and 3' integration correspond to the blue and red arrows displayed in A), respectively, and specific primers used were: ML4158/ML687 (TgNFS2 5' integration), ML1041/ML4159 (TgNFS2 3' integration), ML1774/ML4388 (TgISU1 5' integration), ML1771/ML4387 (TgISU1 3' integration).
