# Supplementary material for: Differential contribution of two organelles of endosymbiotic origin to iron-sulfur cluster synthesis and overall fitness in Toxoplasma
Source: PLoS Pathog. 2021 Nov 18;17(11):e1010096. doi: 10.1371/journal.ppat.1010096 (PMC8639094; doi:10.1371/journal.ppat.1010096)
Supplement: S5 Fig — A) Schematic representation of the strategy for generating TgNFS2 and TgISU1 complemented cell lines by integrating an extra copy of the gene of interest (GOI) by double homologous recombination at the Uracil Phosphoribosyltransferase (UPRT) locus. Negative selection with 5-fluorodeoxyuridine (FUDR) was used to select transgenic parasites based on their absence of UPRT expression. B) Diagnostic PCR for verifying correct integration of the construct. The amplified fragments confirming integration correspond to the red arrows displayed in A), and specific primers used were: ML2866/ML4686 (TgNFS2 integration), ML2866/ML4455 (TgISU1 5’ integration). C) Semi-quantitative RT-PCR analysis from cKD TgNFS2-HA, cKD TgISU1-HA and their respective complemented cell lines grown for three days in the presence or absence of ATc, using specific primers couples ML4686/ML4687 (TgNFS2) and ML4684/ML4685 (TgISU1). It shows complemented cell lines express high levels of the corresponding mRNA. Specific β-tubulin primers (ML841/ML842) were used as controls. (PDF) [file ppat.1010096.s005.pdf]

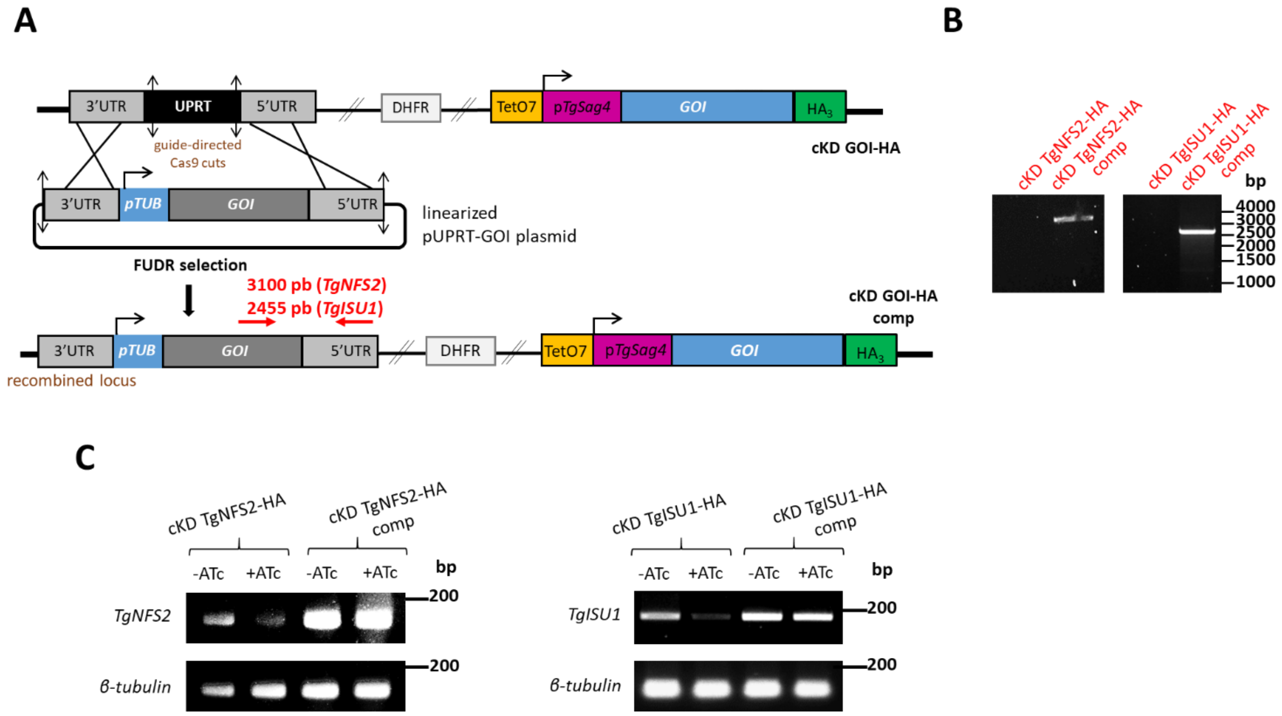

S5 Fig. Generation of TgNFS2 and TgISU1 complemented cell lines. A) Schematic representation of the strategy for generating TgNFS2 and TgISU1 complemented cell lines by integrating an extra copy of the gene of interest (GOI) by double homologous recombination at the *Uracil Phosphoribosyltransferase* (*UPRT*) locus. Negative selection with 5-fluorodeoxyuridine (FUDR) was used to select transgenic parasites based on their absence of UPRT expression. B) Diagnostic PCR for verifying correct integration of the construct. The amplified fragments confirming integration correspond to the red arrows displayed in A), and specific primers used were: ML2866/ML4686 (TgNFS2 integration), ML2866/ML4455 (TgISU1 5' integration). C) Semi-quantitative RT-PCR analysis from cKD TgNFS2-HA, cKD TgISU1-HA and their respective complemented cell lines grown for three days in the presence or absence of ATc, using specific primers couples ML4686/ML4687 *(TgNFS2)* and ML4684/ML4685 (*TgISU1*). It shows complemented cell lines express high levels of the corresponding mRNA. Specific *β-tubulin* primers (ML841/ML842) were used as controls.
