# Supplementary material for: Differential contribution of two organelles of endosymbiotic origin to iron-sulfur cluster synthesis and overall fitness in Toxoplasma
Source: PLoS Pathog. 2021 Nov 18;17(11):e1010096. doi: 10.1371/journal.ppat.1010096 (PMC8639094; doi:10.1371/journal.ppat.1010096)
Supplement: S6 Fig — Percentage of cKD TgNFS2-HA parasites-containing vacuoles displaying a loss of apicoplast signal when labeled with streptavidin-Fluorescein Isothiocyanate after culture in the presence or absence of ATc for 120 hours. Data are mean values from n = 3 independent experiments ±SEM. **** p ≤ 0.0001, Student’s t-test. Inset: typical streptavidin labeling of the apicoplast (green) in a tachyzoites-containing vacuole, the inner membrane complex was stained with anti-TgIMC3 (red) and the DNA with DAPI (blue); scale bar = 5μm. (PDF) [file ppat.1010096.s006.pdf]

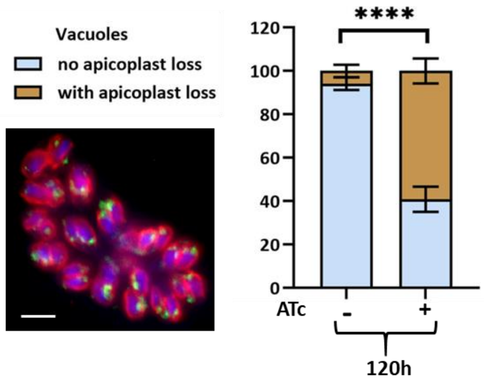

S6 Fig. Quantification of apicoplast loss upon TgNFS2 depletion using streptavidin. Percentage of cKD TgNFS2-HA parasites-containing vacuoles displaying a loss of apicoplast signal when labeled with streptavidin-Fluorescein Isothiocyanate after culture in the presence or absence of ATc for 120 hours. Data are mean values from *n*=3 independent experiments ±SEM. **** $p \leq 0.0001$, Student's *t*-test. Inset : typical streptavidin labeling of the apicoplast (green) in a tachyzoites-containing vacuole, the inner membrane complex was stained with anti-TgIMC3 (red) and the DNA with DAPI (blue); scale bar=5µm.
