# Supplementary material for: Differential contribution of two organelles of endosymbiotic origin to iron-sulfur cluster synthesis and overall fitness in Toxoplasma
Source: PLoS Pathog. 2021 Nov 18;17(11):e1010096. doi: 10.1371/journal.ppat.1010096 (PMC8639094; doi:10.1371/journal.ppat.1010096)
Supplement: S7 Fig — Classification of variant proteins according to their putative cellular localization (A) and function (B). N/A: not available; ER: endoplasmic reticulum; PM: plasma membrane; VAC: vacuolar compartment; GRA: dense granule protein; SRS: SAG-related sequence. In particular, the increased expression of ER-located lipid metabolism enzymes suggests possible compensation for loss of apicoplast-related lipid synthesis function. (PDF) [file ppat.1010096.s007.pdf]

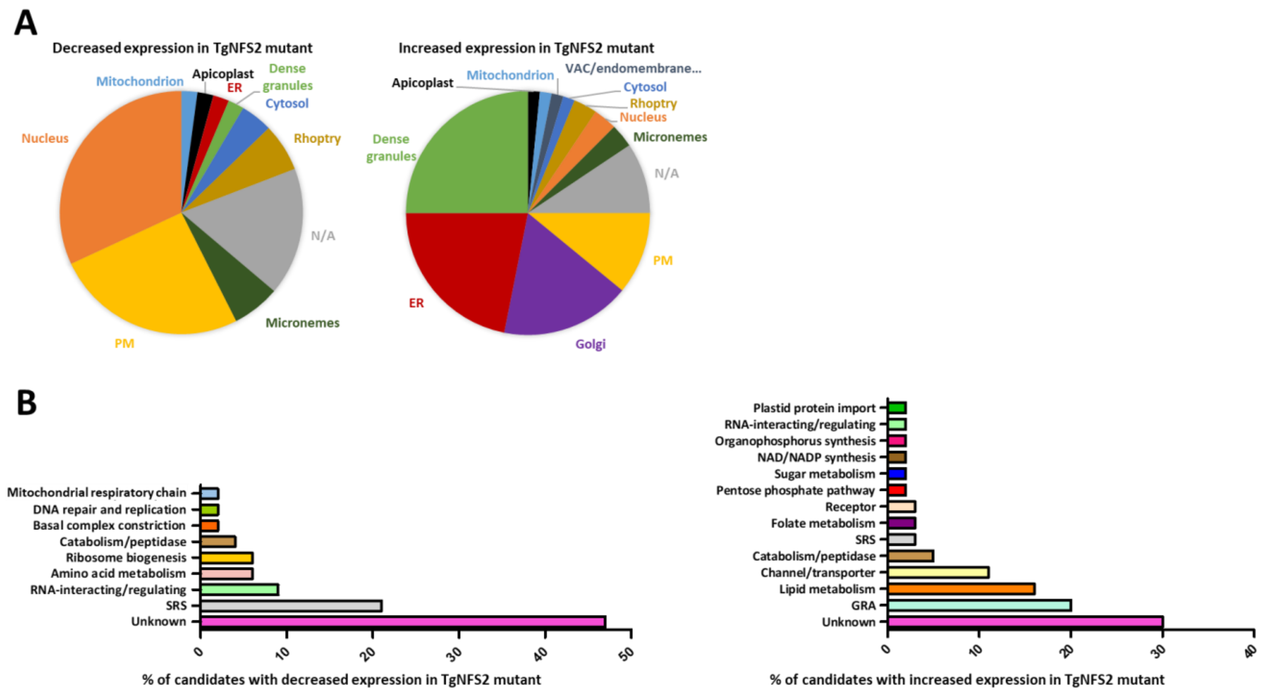

S7 Fig. Quantitative proteomics shows depletion of TgNFS2 does not have a global impact on the apicoplast, but may suggest compensatory response from other cellular pathways in response to specific apicoplast-related lipid synthesis defects. Classification of variant proteins according to their putative cellular localization (A) and function (B). N/A: not available; ER: endoplasmic reticulum; PM: plasma membrane; VAC: vacuolar compartment; GRA: dense granule protein; SRS: SAG-related sequence. In particular, the increased expression of ER-located lipid metabolism enzymes suggests possible compensation for loss of apicoplast-related lipid synthesis function.
