# Supplementary material for: Differential contribution of two organelles of endosymbiotic origin to iron-sulfur cluster synthesis and overall fitness in Toxoplasma
Source: PLoS Pathog. 2021 Nov 18;17(11):e1010096. doi: 10.1371/journal.ppat.1010096 (PMC8639094; doi:10.1371/journal.ppat.1010096)
Supplement: S8 Fig — Classification of variant proteins according to their putative cellular localization (A) and function (B). N/A: not available; ER: endoplasmic reticulum; PM: plasma membrane; VAC: vacuolar compartment; GRA: dense granule protein; SRS: SAG-related sequence. A large proportion of components of complexes II, III and IV of the mitochondrial respiratory chain, which involve Fe-S proteins, were found to be less abundant. Conversely, the abundance of many bradyzoite-specific dense granule proteins of plasma membrane-located surface antigens increased. (PDF) [file ppat.1010096.s008.pdf]

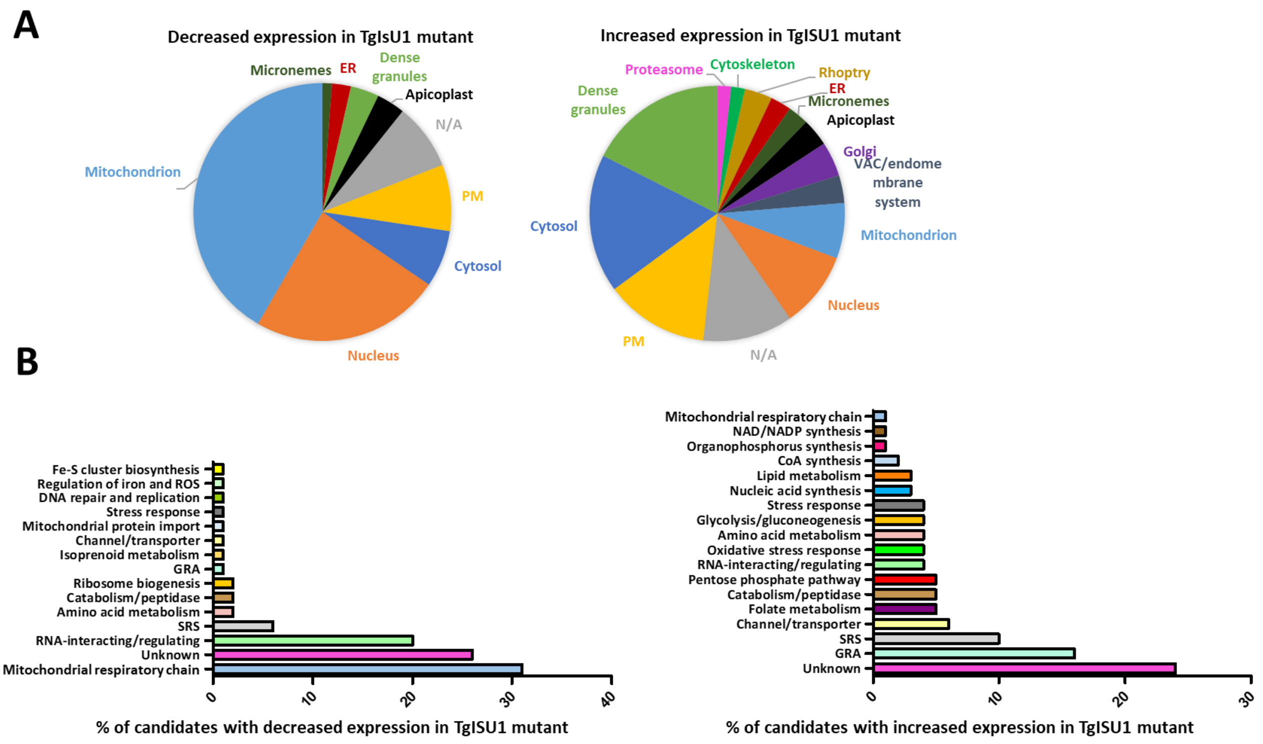

S8 Fig. TgISU1-depleted parasites show a marked decrease in proteins related to mitochondrial respiration, and a strong increase in bradyzoite-specific dense granules proteins and surface antigens. Classification of variant proteins according to their putative cellular localization (A) and function (B). N/A: not available; ER: endoplasmic reticulum; PM: plasma membrane; VAC: vacuolar compartment; GRA: dense granule protein; SRS: SAG-related sequence. A large proportion of components of complexes II, III and IV of the mitochondrial respiratory chain, which involve Fe-S proteins, were found to be less abundant. Conversely, the abundance of many bradyzoite-specific dense granule proteins of plasma membrane-located surface antigens increased.
