# Supplementary material for: Differential contribution of two organelles of endosymbiotic origin to iron-sulfur cluster synthesis and overall fitness in Toxoplasma
Source: PLoS Pathog. 2021 Nov 18;17(11):e1010096. doi: 10.1371/journal.ppat.1010096 (PMC8639094; doi:10.1371/journal.ppat.1010096)
Supplement: S9 Fig — A) Schematic representation of the strategy for generating the conditional knock-down cell line by homologous recombination at the native locus. Pyrimethamine was used to select transgenic parasites based on their expression of Dihydrofolate reductase (DHFR). B) Diagnostic PCR for verifying correct integration of the construct. The amplified fragments confirming 5’ and 3’ integration correspond to the blue and red arrows displayed in A), respectively, and specific primers used were ML1774/ML4388 (5’ integration), and ML1771/ML4387 (3’ integration). C) Semi-quantitative RT-PCR analysis of the cKD TgISU1 cell line grown for up to three days in the presence or absence of ATc, using specific primers couple ML4684/ML4685, showing effcient down-regulation of TgISU1 expression. Specific actin primers (ML843/ML844) were used as controls. D) Plaque assays were carried out by infecting HFF monolayers with the newly generated cKD TgISU1 cell line or the original cKD TgISU1-HA mutant cell line as a control. They were grown for 7 days ± ATc. Measurements of lysis plaque areas are shown on the right and confirm a significant defect in the lytic cycle in the two mutant cell lines upon ATc addition. Values are means of n = 3 experiments ± SEM. **** denotes p ≤ 0.0001, ANOVA. Scale bar = 1mm. (PDF) [file ppat.1010096.s009.pdf]

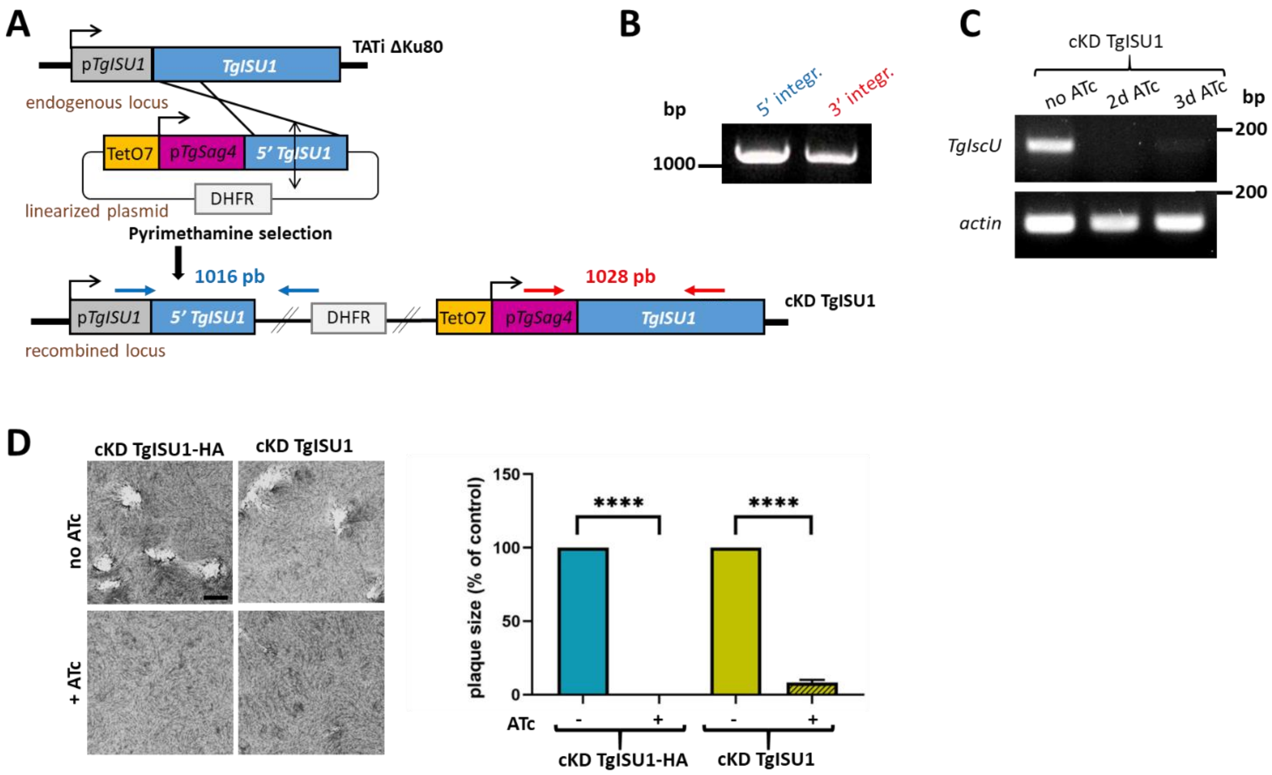

S9 Fig. Generation of a tag-free cKD TgISU1 cell line. A) Schematic representation of the strategy for generating the conditional knock-down cell line by homologous recombination at the native locus. Pyrimethamine was used to select transgenic parasites based on their expression of Dihydrofolate reductase (DHFR). B) Diagnostic PCR for verifying correct integration of the construct. The amplified fragments confirming 5’ and 3’ integration correspond to the blue and red arrows displayed in A), respectively, and specific primers used were ML1774/ML4388 (5’ integration), and ML1771/ML4387 (3’ integration). C) Semi-quantitative RT-PCR analysis of the cKD TgISU1 cell line grown for up to three days in the presence or absence of ATc, using specific primers couple ML4684/ML4685, showing effcient down-regulation of *TgISU1* expression. Specific *actin* primers (ML843/ML844) were used as controls. D) Plaque assays were carried out by infecting HFF monolayers with the newly generated cKD TgISU1 cell line or the original cKD TgISU1-HA mutant cell line as a control. They were grown for 7 days ± ATc. Measurements of lysis plaque areas are shown on the right and confirm a significant defect in the lytic cycle in the two mutant cell lines upon ATc addition. Values are means of n=3 experiments ± SEM. **** denotes $p \leq 0.0001$, ANOVA. Scale bar= 1mm.
