# Supplementary material for: Differential contribution of two organelles of endosymbiotic origin to iron-sulfur cluster synthesis and overall fitness in Toxoplasma
Source: PLoS Pathog. 2021 Nov 18;17(11):e1010096. doi: 10.1371/journal.ppat.1010096 (PMC8639094; doi:10.1371/journal.ppat.1010096)
Supplement: S10 Fig — A) Schematic representation of the strategy for expressing HA-tagged versions of TgSDHB and TgApiCox13 by homologous recombination at the native locus of the corresponding gene of interest. Chloramphenicol was used to select transgenic parasites based on their expression of the Chloramphenicol acetyltransferase (CAT). B) Diagnostic PCR for verifying correct integration of the construct. The amplified fragment corresponds to the red arrows in A), and specific primers used were ML1476/ML5013 (TgSDHB), ML1476/ML5006 (TgApiCox13). (PDF) [file ppat.1010096.s010.pdf]

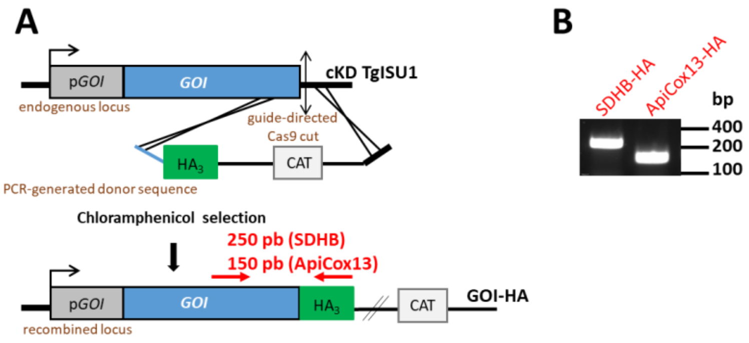

S10 Fig. Tagging of mitochondrial candidates in the cKD TgISU1 background. A) Schematic representation of the strategy for expressing HA-tagged versions of TgSDHB and TgApiCox13 by homologous recombination at the native locus of the corresponding gene of interest. Chloramphenicol was used to select transgenic parasites based on their expression of the Chloramphenicol acetyltransferase (CAT). B) Diagnostic PCR for verifying correct integration of the construct. The amplified fragment corresponds to the red arrows in A), and specific primers used were ML1476/ML5013 (TgSDHB), ML1476/ML5006 (TgApiCox13).
