# Supplementary material for: Differential contribution of two organelles of endosymbiotic origin to iron-sulfur cluster synthesis and overall fitness in Toxoplasma
Source: PLoS Pathog. 2021 Nov 18;17(11):e1010096. doi: 10.1371/journal.ppat.1010096 (PMC8639094; doi:10.1371/journal.ppat.1010096)
Supplement: S11 Fig — A) Stage conversion was induced by alkaline pH stress on type I parasites of the RH Tati Δ80 parental cell line or the cystogenic type II Prugniaud strain for up to 14 days. Fixed samples were co-stained with cyst wall marker DBL, together with tachyzoite marker SAG1, or intermediate (P18/SAG4) or late (P21) bradyzoite markers. Scale bar represents 10 μm. DNA was labelled with DAPI. B) The percentage of DBL-positive cysts containing P18 or P21 staining was evaluated on samples after 14 days of ATc treatment (for TgISU1, TgQCR11 and TgmS35 conditional mutants) or pH stress (for RH TATi ΔKu80 and Prugniaud parasites). Values are mean ±SEM from n = 3 independent experiments. C) Measurement of the cyst area size after growing the cell lines for 7 and 14 days in cyst-inducing conditions, then labelling the cyst wall with DBL and measuring the surface of at least 25 cysts per condition. Values are mean ±SD from three independent biological replicates. * p ≤ 0.05, ** p ≤ 0.01, Student’s t-test, when comparing values after 14 days between the type II Prugniaud strain and the type I cell lines. (PDF) [file ppat.1010096.s011.pdf]

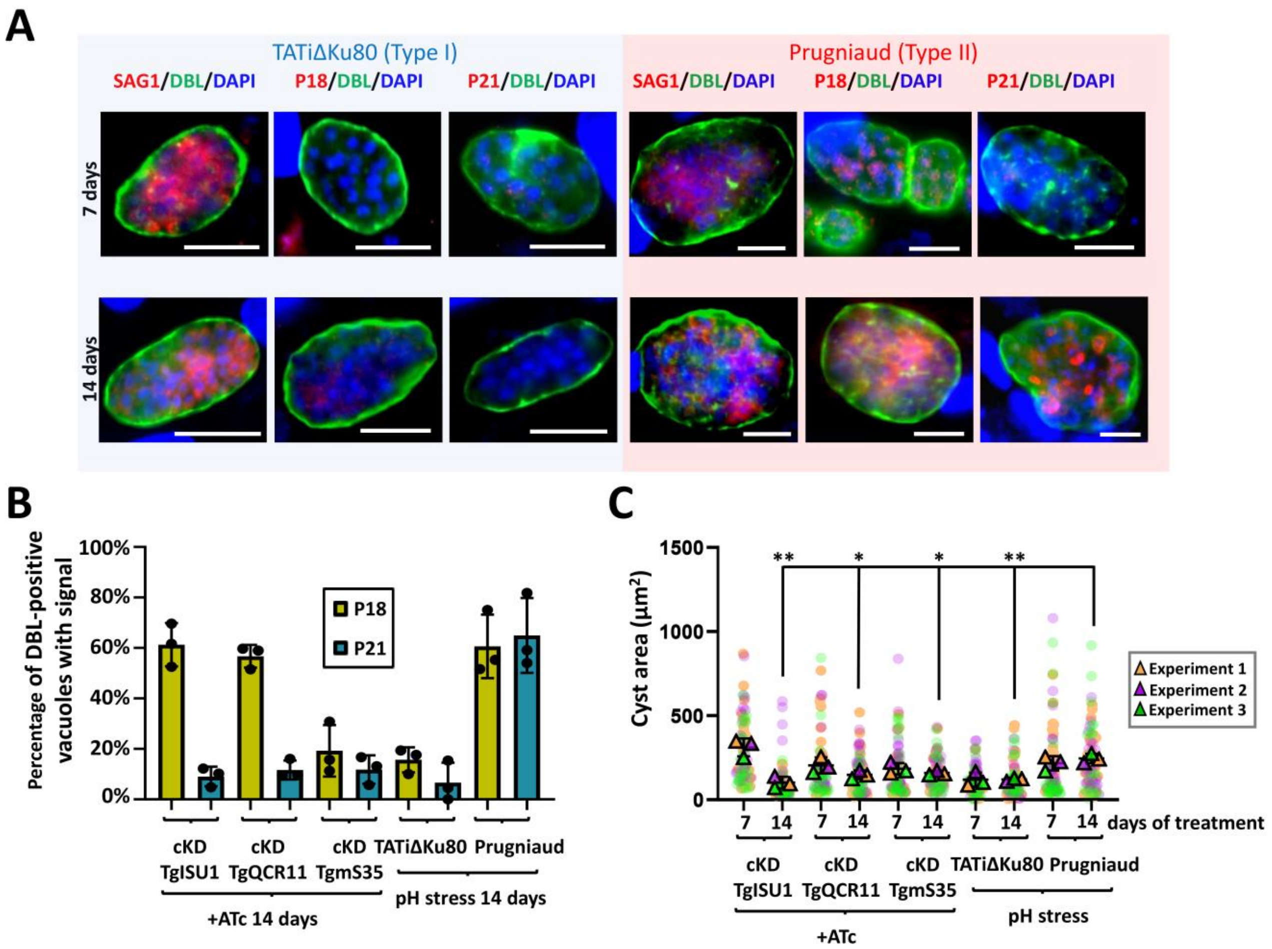

S11 Fig. Comparison of stage conversion for type I and II strains. A) Stage conversion was induced by alkaline pH stress on type I parasites of the RH Tati Δ80 parental cell line or the cystogenic type II Prugniaud strain for up to 14 days. Fixed samples were co-stained with cyst wall marker DBL, together with tachyzoite marker SAG1, or intermediate (P18/SAG4) or late (P21) bradyzoite markers. Scale bar represents 10 µm. DNA was labelled with DAPI. B) The percentage of DBL-positive cysts containing P18 or P21 staining was evaluated on samples after 14 days of ATc treatment (for TgISU1, TgQCR11 and TgmS35 conditional mutants) or pH stress (for RH TATi ΔKu80 and Prugniaud parasites). Values are mean ±SEM from *n*=3 independent experiments. C) Measurement of the cyst area size after growing the cell lines for 7 and 14 days in cyst-inducing conditions, then labelling the cyst wall with DBL and measuring the surface of at least 25 cysts per condition. Values are mean ±SD from three independent biological replicates. * $p \leq 0.05$, ** $p \leq 0.01$, Student's *t*-test, when comparing values after 14 days between the type II Prugniaud strain and the type I cell lines.
