# Supplementary material for: Differential contribution of two organelles of endosymbiotic origin to iron-sulfur cluster synthesis and overall fitness in Toxoplasma
Source: PLoS Pathog. 2021 Nov 18;17(11):e1010096. doi: 10.1371/journal.ppat.1010096 (PMC8639094; doi:10.1371/journal.ppat.1010096)
Supplement: Attachment — Submitted filename: Pamukcu et al response to reviewers.pdf [file ppat.1010096.s019.pdf]

Thanks for taking the time to evaluate our manuscript. Please find below a point-by-point response to the comments.

Part II – Major Issues: Key Experiments Required for Acceptance
Please use this section to detail the key new experiments or modifications of existing experiments that should be absolutely required to validate study conclusions.

Generally, there should be no more than 3 such required experiments or major modifications for a "Major Revision" recommendation. If more than 3 experiments are necessary to validate the study conclusions, then you are encouraged to recommend "Reject".

Reviewer #1: None. The revision has addressed the points raised by me.

Thanks.

Reviewer #2: 1. Figure 9D and Figure 10D. The experiments here present single images of P18-positive and SAG1-depleted vacuoles. Given the uncertainties in the data about whether the slower growing vacuoles are ‘real’ tissue cysts, these would benefit from quantification (i.e. what percentage of parasite vacuoles are P18-positive following TgISU1 knockdown? Does this mimic the observations of the DBL-positive vacuoles in Fig 9B/10C?). Alternatively, the authors could consider undertaking western blotting to determine whether the various marker proteins are up/down-regulated upon gene knockdown.

Thank you for your comment. First of all, a clarification concerning the in vitro-derived bradyzoites in general: there is no such thing as a “real” cyst when using in vitro differentiation systems. In fact, several transcriptomic studies have shown that in vitro-derived bradyzoites are somewhat intermediate between tachyzoites and in vivo-derived mature tissue cysts, but they certainly cannot be considered as “real” cysts. There is no perfect system for generating bradyzoites in vitro, and although models with different host cell types are currently being optimised (see https://doi.org/10.1101/2021.01.15.426845 and https://doi.org/10.1098/rsob.210053 for recent examples), working with in vitro differentiation assays remains quite challenging.

To be more precise, when performing in vitro differentiation assays the first issue is the lack of synchronisation in the differentiation process, as illustrated below by this IFA on 14 days in vitro cysts showing heterogeneity in size and in SAG1/P30 labelling intensity.

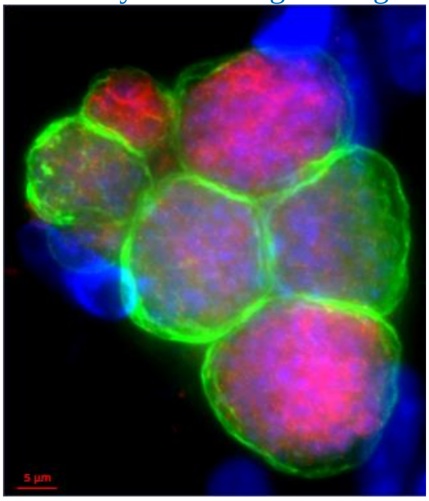

TgISU1 parasites kept in the presence of ATc for 7 days and co-stained with DBL to label the cyst wall (green) and P30/SAG1 (red)

The second issue is that not all parasites convert as efficiently, and as a consequence tachyzoites remain in culture and contribute to the heterogeneity of the observed phenotypes, as illustrated below by this IFA on 14 days in vitro cysts showing a cyst surrounded by actively replicating tachyzoites.

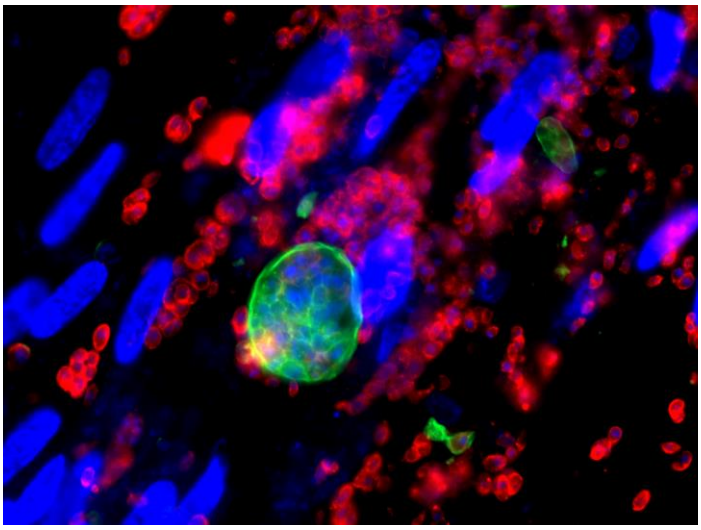

TATi Δku80 parasites kept for 14 days under alkaline stress conditions showing a mature DBL-labelled cyst (in green) surrounded by P30-labelled tachyzoites (red)

For these reasons, it is difficult to undertake comparative quantitative, and especially immunoblot quantifications for example, on in vitro-derived bradyzoites analyses after long term stage conversion.

The data presented on Figure 9B/10C which are mentioned by this reviewer, have been obtained for early timepoints (up to 48hours), when it is still relatively simple to monitor conversion. However, in order to reply to this comment and obtain quantitative data for later timepoints (7 days, and up to 14 days), we have chosen to quantify the presence of P18 and P21 signals in DBL-positive cysts (regardless of their size or staining intensity). This is now presented in a new Figure S11. These numbers clearly support the representative images we have chosen to illustrate Figures 9 and 10.

2. Figure 9D and Figure 10D. Given that the authors are working with RH/TATi-background parasites that typically don't readily differentiate to bradyzoites, they should also induce bradyzoite differentiation using a more standard approach (e.g. NO treatment as in Figure 9B) to determine whether markers like P18 and P21 'turn on' in their parasite strain. This would test whether the observation that "stage conversion of these parasites progresses beyond the appearance of early cyst wall markers … but it seems incomplete" (Lines 441-443) is specific to the TgISU1/TgQCR11 mutants, or because the parental strain doesn't readily undergo switching to bradyzoites.

Thanks for this suggestion, this can be a useful reference for the cyst markers we have used in the study. Thus, we have now included differentiation data not only for the RH TATi Δku80 type I parental cell line, but also for the type II Prugniaud strain as a positive control. On these cell lines we have used alkaline stress-induced differentiation in absence of CO_2 (which is the most common approach for in vitro bradyzoite induction) and managed to keep them for up to 14 days. This was challenging for the type I parasites, which have a low conversion rate and had to be inoculated at low concentration (as now mentioned in the 'Materials and Methods'

section). Again, we focused on the DBL-positive cysts and quantified P18 and P21 staining at 14 days, as well as cyst size at 7 and 14 days (now presented on Figure S11). Our results confirm the RH TATi Δku80 parasites do not convert efficiently and hardly express any of the intermediate or late bradyzoite markers we have tested in this study. With this regard, TgISU1 and TgQCR11 thus seem to commit more to differentiation than the parental cell line. Also, all type I strains displayed smaller cyst size than the type II parasites after long term induction of stage conversion, suggesting incomplete conversion and subsequent reactivation likely happens.

Part III – Minor Issues: Editorial and Data Presentation Modifications
Please use this section for editorial suggestions as well as relatively minor modifications of existing data that would enhance clarity.

Reviewer #1: Line 108-109: ""... investigations in apicomplexan parasites have been so far almost
exclusively focused on the apicoplast-located SUF pathway [26–30] and mostly in Plasmodium
species." There's a recent publication which includes structure-function analyses of the P. falciparum mitochondrial ISC pathway (Sadik et al. 2021, Molecular Microbiology - https://doi.org/10.1111/mmi.14735). This should be cited.

It is now cited.

Reviewer #2: 1. Line 35-36. "our data highlighted distinct changes at the metabolic level." The study does not undertake any metabolomics, so this statement isn't supported by the data. It would be truer to state that changes occurred in the abundance of proteins from various metabolic pathways, suggesting changes at the metabolic level. Similarly, later in this paragraph: "the mitochondrial pathway is clearly crucial for maintaining their respiratory capacity." I don't disagree that this is a likely possibility, but the study measures respiratory capacity indirectly (by measuring membrane potential). Some caution is warranted here: "the mitochondrial pathway likely supports the maintenance of respiratory capacity" or similar.

We are now less affirmative and use conditional statements as suggested.

2. Figure 1B. What is the statistical test comparing? IscU/SufS mutants vs the strains complemented with the Toxoplasma homologues? Just the ones cultured in the absence of the chelator? Please clarify this.

It compares mutant vs complemented strains in the absence of chelator. This is now mentioned in the figure legend.

3. Line 242-243. "The growth kinetics we observed for this mutant are consistent with the "delayed death" effect observed in apicoplast-defective parasites". It isn't clear what "growth kinetics" are being referred to here? Is it that growth of the NFS2 mutant was "slowed considerably" following the second invasion cycle (Line 236)? This isn't quantified in any way. e.g. Figure 4C is presumably measuring growth in the second cycle, but there is no measure of the first cycle from what I can tell. Please clarify.

As shown on figure 5A, TgNFS2 mutant parasites are initially slowed down but can egress eventually and reinvade before being completely blocked, which is reminiscent of the "delayed death" phenotype. This is now clarified in the text.

4. Line 424-425. "This clearly confirmed a strong increase in the expression of bradyzoite-specific SRS in the TgISU1 mutant". This makes it seem like bradyzoite-specific SRS proteins are upregulated across the board, whereas this is only true of one (SRS44, which is also upregulated in the NFS2 mutant) or maybe two (SRS13). This suggests that the "differentiation" that the authors are observing is different to typical bradyzoite differentiation. These experiments should therefore be described with more precision (i.e. noted that some but not most bradyzoite-specific SRSs are upregulated upon ISU1 knockdown).

We agree SRS44 and SRS13 are the one which are the most abundant, but overall it is striking that most SRS proteins that have positive (albeit more modest than for the two aforementioned SRS) enrichment values are more expressed in bradyzoites. It should also be noted that transcriptomics data available on stage conversion has been classically obtained on type II strains and some strain specificities may also account for some of these differences. Nevertheless, as suggested by this reviewer, we now mention not all bradyzoite-specific SRSs are upregulated and that it may reflect an atypical or incomplete stage conversion.

5. Line 441-443. "stage conversion of these parasites progresses beyond the appearance of early cyst wall markers, but not only it does so with slow kinetics, but it seems incomplete." The meaning here isn't entirely clear. Perhaps "… but does so with slow kinetics and seems incomplete." Also need to specify what is meant by "slow kinetics" - slower than what? As noted in the major comments, using a parasite strain that has been induced to form bradyzoites using standard approaches would allow for a more relevant comparison.

We do not mention "slow kinetics" anymore and only refer to an incomplete conversion, by comparison with the type II control that is now included and shows expression of late bradyzoite markers like P21 (Figure S11).

6. Lines 532-534. "the three putative rhoptry localized candidates significantly less expressed in the TgNFS2 mutant (Table S3) are predicted to be GPI-anchored and/or glycosylated.". Based on what evidence? Please provide relevant references that these are GPI anchored/glycosylated.

Predictions were done using specific webservers described in the 'Materials and Methods' section and whose references are now mentioned in the text.

7. Line 542. "long term depletion of TgISU1" – TgNFS2?

Thank you for spotting this, it has been corrected.

8. Line 580-581. "perhaps because of their metabolic flexibility, we have shown that mutant parasites seem to retain the ability to survive as tachyzoites, or initiate a switch to bradyzoites." The first point (survival as tachyzoites) reproduces previous findings in the

TgQCR11 mutant parasites by Hayward et al (ref 61).
This is now mentioned.

9. Line 602. “the ETC mutants (TgQCR11 and TgISU1)”. ISU1 is not an ETC mutant.

It has been changed to “Also, contrarily to the mutants where the ETC may be more directly impacted (TgQCR11 and TgISU1),…”

10. Line 603-603. “largely continued to thrive”. “Thrive” would imply that the parasites are growing well, which I don’t think the authors mean to imply.

Yes, we changed that part to “then continued to multiply to some extent, and lyse their host cells…”.
