# Supplementary material for: Differential contribution of two organelles of endosymbiotic origin to iron-sulfur cluster synthesis and overall fitness in Toxoplasma
Source: PLoS Pathog. 2021 Nov 18;17(11):e1010096. doi: 10.1371/journal.ppat.1010096 (PMC8639094; doi:10.1371/journal.ppat.1010096)
Supplement: Attachment — Submitted filename: Rebuttal Pamukcu et al.docx [file ppat.1010096.s018.docx]

Thanks to all reviewers for a very thorough examination of our manuscript. They will find a point-by-point reply to your comments below. We took great care in addressing all the points that were raised. As suggested, the revised version of the manuscript includes additional controls and experimental validation of our hypotheses, that we think definitely improves the paper.

Of note, as suggested by one of the reviewers, we are now adopting a plant-like nomenclature in the manuscript (TgISCU became TgISU1 and TgSUFS became TgNFS2), while retaining the original names in this rebuttal for the sake of clarity.

Part I - Summary

Reviewer #2:

There is room for better survey of the existing literature on ISC in Toxoplasma organelles in particularly the recent paper on CIA from Aw et al 2020 which is only mentioned a couple of times in passing despite being the most relevant Toxoplasma work.

The Aw et al. paper came out in the late stages of preparation of our own manuscript. As suggested, we are now mentioning and discussing this paper more, establishing a parallel between our mutant and the TgNFS1 mutant and the implications of ISC disruption on SDHB down-regulation and on the mitochondrial electron transport chain (see l.106-107, l.394-397, l.565-567, l.589-590).

At first the authors identify all the potential components via homology searches and characterise them using the available data from previous work (here is good example of mentioning previous work that already identified some components).

The author then uses an elegant bacterial complementation system to provide support for the activity of the putative Toxoplasma SufS and IscU. In these assays it would be important to include the bacterial wild type complementation to understand the level of rescue.

Absolutely, we agree the wild-type control should have been included in the first place. We have now included growth data for the parental *E. coli* strain as a control in a new Figure 1B. Complemented strains have a slightly different growth kinetics and are more sensitive to iron depletion (hinting a potentially intermediate complementation of the phenotype), but they reach similar bacterial densities to the parental line in stationary phase, contrarily to the mutant strains.

Next the authors use endogenous tagging and immunofluorescent assay (IFA) to validate the localization of SufS and IscU to the mitochondrion and plastid, as well as one of the candidate SufE proteins. Following this validation, and through the use of inducible promoter, the authors generated regulatable mutants, and their corresponding complemented lines, which are used to show the specific outcome of TgSufS or TgIscU depletion on parasite replication and growth. These data are mainly solid. It is important however to look at expression level in a technically and statistically valid way that allows demonstration of similar levels of down-regulation (e.g. qRT-PCR) to exclude this as reason for the different phenotypes.

We agree that comparing knock-down mutants is sometimes not an easy task. In fact, even similar levels of down-regulation do not guarantee same levels of phenotypic impact for independent protein targets. In our case, both mutants have very different phenotypes, and for each one we already provide demonstration by independent experimental approaches that down-regulation is tight and efficient. First, we show by immunoblot and IFA that the IscU and SufS proteins are depleted in an efficient way. Second, in parallel we used semi-quantitative RT-PCR analysis of mRNA levels for both mutant and complemented cell lines (Figure S5C). Finally, these complemented cell lines fully support phenotype specificity (and perhaps it should be noted that many Toxoplasma studies do not even make a specific effort in providing functional complementation for conditional knock-down mutants). In conclusion, it seems clear that the phenotypes of the two mutants are very different and this likely has a biological explanation rather than a link with hypothetical minute differences in residual amounts of the proteins of interest; thus we think quantification by qRT-PCR is unnecessary.

Regarding the observed phenotype, a relevant point of notice is that the two genes studied are not equivalent points of the pathway: one is the initial cysteine desulfurase while the other is the initial scaffold. Seeing that this is not a “like-for-like” comparison the description of the different phenotypes throughout the manuscript should highlight this context. Likewise it might be helpful for the authors to explain why they didn’t choose to study IscU and SUFB, which would be more comparable.

It is widely described in the literature (and accepted in the Fe-S research community) that interfering with the function of the upstream machinery such as the desulfurases, or the scaffold proteins, leads to a comparable disruption of the whole Fe-S cluster biogenesis pathway. This is now mentioned in the manuscript on l.145-151.

Of course this would have been a completely different outcome if we had chosen to interfere with the function of more downstream effectors such as transfer proteins, which have a distinct repertoire of client proteins and whose disruption likely has different consequences at the cellular level.

We initially tried to target several of these key upstream components with various success and, quite simply, the SufS and IscU mutants were the cell lines that we first managed to obtain. Based on experimental investigation in other eukaryotic models, they seem perfectly valid to assess the general function of the plastidic and mitochondrial Fe-S cluster biogenesis pathways.

Of note, we more recently managed to obtain the SufC mutant (part of the scaffolding complex in the SUF pathway, and thus equivalent to IscU function in the ISC pathway). Although we did not perform the quantitative proteomics analysis on this particular mutant, our preliminary data shows it recapitulates phenotypically the growth defect and apicoplast-related impact (including default in lipoylation of the PDH E2 subunit) that we described in the SufS mutant (Pamucku et al, unpublished).

The effect of depletion on organelle morphology/presence is then evaluated by IFA. While the apicoplast signal with the selected marker is affected, this is not direct evidence for apicoplast loss. This could be further supported by qPCR for apicoplast genome (or at least this caveat should be highlighted). Further, the suggested no-effect on mitochondrial morphology is not well supported – a careful evaluation of morphology range (e.g. as done here - PMID: 28202940) would provide stronger support.

It is true using a single apicoplast marker does not completely prove the disappearance of the organelle. Not only are we now mentioning this in the manuscript (l.238-242), but we also provide additional quantification of apicoplast loss using a second independent marker, as now described in a new supplemental figure (Figure S6).

The absence of visible effect on the mitochondrion was not only evaluated by fluorescence microscopy, but also at the ultrastructural level by electron microscopy. Please see below a representative picture of the IscU mutant kept for 5 days in the presence of ATc and displaying seemingly normal subcellular structures, including mitochondrial membranes and cristae.

Label-free quantitative proteomics is used to demonstrate impact of the depletions on protein stability or expression. These experiments should include a control and should be interpreted with more care. The authors makes an assumption that Fe-S addition has a role in proper folding and thus that disruption of the organelle specific pathway would affect stability and/or expression. Firstly, it would be appropriate to cite the existing support for this (Aw et al – down-regulating NFS1 caused decrease in SdhB protein).

Our initial hypothesis that disrupting Fe-S clusters synthesis would affect the abundance of Fe-S proteins is not based on the Aw et al paper which, as mentioned above, came out after we completed our quantitative proteomics analysis and most of the experiments performed for the first version of the present paper. Instead, it comes from our own extensive experience of quantitative proteomics of Fe-S mutants in plant models (please see [10.1093/jxb/eraa166](https://doi.org/10.1093/jxb/eraa166) and [10.3390/ijms21218121](https://doi.org/10.3390/ijms21218121) as recent examples). That being said, it is true that the findings of Aw et al that ISC pathway disruption affects stability of SDHB protein are similar and we are now mentioning and discussing this in more details (l.394-397, l.565-567).

Importantly, to provide support for a specific effect on protein stability in the case of the herein describes mutant the proteomics for each mutant should be compared to a mutant of a non-Fe-S biosynthesis protein in the respective organelle.

The same comment applies for the specific observation of the seeming activation of expression of stage conversion proteins in the mutant – how do these changes compare to changes seen in other mutants, and what data suggests these observation are specific to Fe-S synthesis mutants over any stress-inducing mutants, or indeed, any mutant. Comparison to controls such as the above mentioned would provide critical support to this interpretation. Equally this is an issue in the interpretation of the observation that not only three Fe-S cluster containing mitochondrial proteins have their expression affected by TgIscU depletion, but also other components of the mETC complexes. True, this could be related to effect on Rieske and ApiCox3, but it could also be an outcome of a general mitochondrial defect. Here again comparison to another unrelated mitochondrial mutant would provide support to specificity. The observation that mitochondrial respiratory defect in this mutant coincide with up-regulation of glycolytic enzymes is interesting and provides exciting support to other observations in the field supporting the alternative pathways for energy conversion used by the parasite. Again, this could be a hallmark of a variety of mitochondrial mutants, which a control might clarify.

This reviewer is asking for quantitative proteomics analyses of other, unrelated mitochondrial mutants which, although potentially insightful about how a particular protein defect would translate into a specific phenotype in the organelle, is a considerable task that goes way beyond the scope of the present study.

Our proteomic study was performed early after depletion of the IscU protein to avoid observations that would stem from a more general mitochondrial defect. In our proteomics dataset, we detected peptides corresponding to 92% of the LOPIT-predicted mitochondrial membrane and soluble proteomes. Yet, only 12% of these were found to be significantly less expressed in the IscU mutant. To give a more specific example, as a comparison, enzymes of the mitochondrion-housed part of the heme synthesis pathway protoporphyrinogen oxidase (TGGT1_272490), ferrochelatase (TGGT1_258650) and 5-aminolevulinic acid (ALA) synthase (TGGT1_258690), all of which had several peptides detected in our analysis, were not found to change significantly in abundance upon IscU depletion. All of this argues against a general demise of the organelle and non-specific effect on the mitochondrial electron transport chain and is now mentioned in the manuscript (l.357-362).

On the other hand, 60% of the down-regulated proteins are known members of the mitochondrial respiratory chain. Our systematic annotation revealed that these not only include known Fe-S proteins (Fe-S subunit of the succinate dehydrogenase complex –SDHB-, Rieske protein and TgApiCox13), but also 60% and 82% of the components of complexes III and IV, respectively (including recently characterized parasite-specific subunits [10.1371/journal.ppat.1009301](https://doi.org/10.1371/journal.ppat.1009301)). As shown by others in Toxoplasma ([10.7554/eLife.38097](https://doi.org/10.7554/elife.38097) , [10.1371/journal.ppat.1009211](https://doi.org/10.1371/journal.ppat.1009211), [10.7554/eLife.38131](https://doi.org/10.7554/elife.38131) , [10.1371/journal.ppat.1009301](https://dx.doi.org/10.1371%2Fjournal.ppat.1009301)), depletion of selected members of an mETC complex can result in stalled assembly or impaired stability of the whole complex. This is now mentioned in the manuscript (l.369-372).

As for conducting a specific joint proteomic and metabolic study of several mitochondrial electron transport chain mutants to get insights on metabolic adaptation of tachyzoites to the lack of mitochondrial ATP production, again although we completely agree this would be very interesting to do. Yet we hope the reviewer will realise it is a whole research project on its own that goes far beyond the scope of the present study.

Additionally, to support the specificity of phenotype it would be relevant to include an examination of an expected affected protein such as rieske.

As suggested, we tagged two candidates belonging to the mitochondrial electron transport chain and highlighted by the proteomics analysis (TgSDHB and TgApiCox13), to verify their change in abundance by immunoblot and immunofluorescence assay. The two proteins were found to be down-regulated, in accordance with the proteomics data. This is now displayed on a new Figure 8.

The point about mitochondrial mutants control becomes even more important later in the manuscript, with regard to the data presented in the final result paragraph, which suggests a link between TgIscU depletion and stage conversion. It is very possible, that any mitochondrial biogenesis and/or functional defect would result in differentiation due to stress. It is important to address this point, and it is doable too, as many mitochondrial mutants are available in the field and could be analysed in the same way as presented here in figure 10. In our opinion this is necessary.

We agree the question whether or not any injury to the mitochondrion can trigger differentiation is a point that deserved further investigation. We thus used the TgQCR11 (a mutant of the complex III of the mitochondrial electron transport chain) and TgmS35 (a more general mutant affected in mitochondrial translation), both kindly provided by this reviewer’s lab, to see if the depletion of these proteins was triggering differentiation too. We found these mutants to be slowed down in growth, but reversibly affected by the depletion of the corresponding proteins. For the TgmS35 mutant, reversibility was even more pronounced than for the TgIscU mutant.

We also found they all initiated stage conversion (based on *Dolichos Biflorus* lectin labelling) upon depletion of their respective proteins, yet they did not progress to full differentiation based on specific bradyzoite marker labelling. In particular, keeping TgmS35 parasites for extended periods of time in culture in the continuous presence of ATc did not prevent lysis of the host cells, suggesting they are able to thrive as tachyzoites. The TgQCR11 mutant behaved quite similarly to the TgIscU mutant.

So, clearly, impacting mitochondrial function does not seem to be lethal for the parasites, which seem to be able to initiate stage differentiation to some extent, even if mutants of the mitochondrial electron transport chain can commit to stage conversion more efficiently than the TgmS35 mutant. However, 1) as stated above, comparing conditional mutants is sometimes not an easy task due to potential differences in residual protein or direct vs indirect phenotypic outcomes, and 2) all these mutants have been generated in a type I strain background, which does not differentiate to bradyzoite spontaneously. Generating mitochondrial mutants in a type II cell line may be much more informative to investigate the impact of mitochondrial defects on stage differentiation. This is now discussed in the manuscript (l.615-616).

Finally, the interpretation about partial stage conversion and potential reactivation events solely based on weak dual staining and vacuole size might benefit from a more careful discussion - lines 409-413 at the moment the topic suffers from extrapolations in our view.

We do not believe our characterization of stage conversion is based on a particularly ‘weak’ analysis. On the contrary, where many studies use *D. biflorus* lectin to detect the increase in abundance of the sole CST1 protein to the nascent cell wall, ours in addition provided quantitative proteomics-based evidence that stage conversion is initiated in the IscU mutant, and also involved the use of antibodies against stage-specific markers. Specifically, it is because we took care to use protein markers of differentiation (whose expression is differentially regulated in time), that we could further postulate this conversion is not complete in the IscU mutant. That being said, stage conversion in type I parasites is known not to progress to the same extend as for type II parasites, so again this should be better investigated in a similar mutant generated in a type II background.

--------------------

<b>Part II – Major Issues: Key Experiments Required for Acceptance<*/b></br>Please use this section to detail the key new experiments or modifications of existing experiments that should be <u>absolutely</u> required to validate study conclusions.<br/><br/>Generally, there should be no more than 3 such required experiments or major modifications for a "Major Revision" recommendation. If more than 3 experiments are necessary to validate the study conclusions, then you are encouraged to recommend "Reject".<br/*><br/>

Reviewer #1: 1.	Lines 315 to 318: “Surprisingly, few apicoplast proteins were impacted. This could reflect a limited impact on apicoplast Fe-S apoproteins, but this is in contradiction with the strong and specific effect we see on the organelle in the absence of TgSufS”. The product of the Isoprenoid biosynthesis pathway is an essential metabolite contributed by the apicoplast; apicoplast loss and subsequent parasite death seen in the TgSufS cKD line might primarily result from the impairment of Fe-S assembly on IspG and IspH. The levels of these proteins are only marginally affected in cKD TgSufS-HA and indirect clues are cited to conclude that this pathway is affected. The authors should directly address whether loss of function of MEP pathway components might be the reason for apicoplast loss. This may be tested by attempting chemical rescue of cKD TgSufS-HA parasites with IPP in the presence or absence of ATc to see whether parasites can overcome delayed death.

Apicoplast rescue by IPP cannot not be tested in *Toxoplasma* because, contrarily to *Plasmodium*, this molecule does not permeate through parasite membranes (see [10.1084/jem.20110039](https://doi.org/10.1084/jem.20110039) ). Others have described a complementation of part of the isoprenoid synthesis pathway by isoprenoid intermediate geranylgeraniol (see [10.1371/journal.ppat.1003665](https://doi.org/10.1371/journal.ppat.1003665) ), so we used it to see if it could complement at least partially the growth phenotype of the TgSufS mutant. However, as shown in the attached figure, it was not the case. Yet, as geranylgeraniol proved to be somewhat toxic to host cells when used at the concentration used in the aforementioned paper, we did not include this in our manuscript. So unfortunately, we are lacking the tools available for *Plasmodium* to assess if alteration of the apicoplast-hosted isoprenoid synthesis pathway is the main reason for the loss of viability of the TgSufS mutant.

Plaque assay was performed on the TgSufS mutant by incubating the parasites for 7 days in the presence or not of ATc and geranylgeraniol

We are currently analysing in more details the death phenotype associated with TgSufS depletion, but we believe it deserves a full analysis as a separate research project, and is thus beyond the scope of the present paper.

Reviewer #2: These points are described above in more detail and here is their summary:

1. better support for the putative function in ISC synthesis

1.a. wild type control for bacterial complementation assay

1.b. include examination of proteins predicted to be affected by ISC synthesis defect (e.g. rieske in the case of the mitochondrion)

We are now providing those controls. See our detailed responses above.

2. inclusion of careful controls for quantitative proteomics via comparison to mutants of a non-ISC biosynthesis protein in the respective organelle

We did not perform quantitative proteomics on unrelated mitochondrial mutants, but we now provide additional information highlighting the specificity of the impact of IscU depletion on the mitochondrial electron transport chain. See our detailed responses above.

3. inclusion or control for the suggested a link between TgIscU depletion and stage conversion

We have now included other mitochondrial mutants in our stage conversion analysis. See our detailed responses above.

Reviewer #3: 1.	Figure 1B. In the bacterial complementation experiments, to what extent does expression of the T. gondii homologues restore bacterial growth? A WT control strain (i.e. non-knockout or KO strain complemented with the bacterial enzyme) is missing from these experiments. Without a WT control, it is difficult to ascertain how much bacterial growth is normally impacted by the iron chelator, or how much less (or more) the mutant and complemented bacteria grow relative to WT, or to what extent complementation rescues the growth defect. Confidence in these data would also be improved with evidence that the TgSufS and TgIscU proteins are being expressed in the bacteria.

We agree we should have included these controls in the first place. We are now providing the growth kinetics of wild-type bacteria as a control. Complemented strains have a slightly different growth kinetics and are more sensitive to iron depletion (hinting a potentially intermediate phenotypic complementation), but they reach similar bacterial densities to the wild-type line in stationary phase, contrarily to the mutant strains.

2.	Figure 4E. The washout experiment is missing controls. It is, for example, unclear whether the (small) plaques observed in the TgIscU-HA washout condition reflect a restoration of growth in the 4 days post-washout, or whether these small plaques would have formed even if ATc had not been washed out. The authors should include a control in which parasites are maintained in ATc for the duration of the experiment (such an experimental condition would also allow for a more appropriate statistical comparison of whether the washout enables a restoration of parasite growth – i.e. rather than the current comparison of the IscU vs SufS mutants) . The 4 day washout period also seems like a very short time to allow plaque formation to occur. The observed restoration may be more apparent if parasites were allowed to grow for longer following washout. The experiment would also benefit from more timepoints for the initial incubation of ATc (i.e. less than 7 days). Presumably the

inability to restore parasite growth in the SufS mutant results from the loss of the apicoplast upon SufS knockdown; additional timepoints would enable the authors to correlate the inability to restore growth in this experiment to the gradual loss of the apicoplast depicted in Figure 5B.

As suggested 1) we have used a longer washout period (7 days instead of 4) and 2) we have included a control with continuous presence of ATc which confirmed the specificity of plaque formation (new Figure 4E).

Regarding the last point, it would be difficult to perform a plaque assay with less than 5-7 days of ATc incubation, as you have to let the parasites go into enough successive lytic cycles to develop visible plaques. Of note, our replication assay, which corresponds to 72h of ATc incubation (48h of pre-incubation +24h), shows growth of the SufS mutant is already affected at this timepoint (Figure 4C), while the apicoplast loss develops much later (Figure 5B). Long term TgSufS-depleted parasites also seem to display general membrane/IMC defects before complete apicoplast loss (see new Figure 7B), suggesting a specific pathway from the organelle, rather than global demise of the apicoplast, would be involved in the lethal phenotype. We are currently analysing this in more details, and it will be the subject of a future study.

3.	The global proteomics approach using in Figure 6 (and elaborated on in Figures 7 and 8) is interesting and potentially powerful. The observation that some proteins appear to increase/decrease in abundance upon knockdown of the candidate FeS synthesis proteins could be indicative of some of the functional roles of the FeS synthesis pathways. Some of this makes biological “sense” (e.g. depletion of respiratory chain proteins, which rely on FeS clusters, in the IscU knockdown). Overall, however, the effects are not huge (although the authors could provide clearer specifics here about exactly which proteins are increased/decreased, and the fold increase/decrease of notable proteins, rather than relying largely on GO terms. Given the global nature of the proteomic data, the conclusions that these differences are real would benefit from a targeted approach that examines specific proteins that are increased/decreased in the proteomic analyses. The authors should epitope tag candidate proteins that appear to increase or are depleted in proteomic analysis of the knockdown mutants and determine at an individual protein level whether these proteins are truly increased/depleted (e.g. proteins in the ETC upon IscU knockdown, lipid synthesis proteins in the ER upon SufS knockdown).

We have made a particular effort to better present the proteomics data in a more informative way (including visualising better fold enrichment and localisation of selected protein candidates), as displayed now on new Figure 6.

We agree epitope-tagging of candidates to verify the trends suggested by the proteomics would be insightful. Unfortunately, our conditional knock-down cell lines already used up the most efficient selectable markers (chloramphenicol was first used to generate a tagged line, and pyrimethamine for replacing the endogenous promoter).

We first tried editing cKD TgSufS-HA and cKD TgIscU-HA parasites with a CRISPR-based approach using the Ble marker, but to no avail. We then decided to generate new untagged cKD TgSufS and cKD TgIscU mutants in the TATi ΔKu80 background. We did not manage to do so in a reasonable timeframe for TgSufS, but we were successful for TgIscU (see Figure S9). In this new cKD TgIscU mutant we efficiently tagged SDHB and TgApiCox13 and could verify by immunoblot and immunofluorescence assay that they are less expressed upon IscU depletion (new Figure 8B, C).

4.	Figure 7C. The authors show that TgSufS knockdown leads to impairment of PDH-E2 lipoylation, and argue that this is because lipoylation requires a functional apicoplast FeS synthesis pathway. However this is an indirect assay, and the data could also be explained by TgSufS leading to a depletion of the TgPDH-E2 protein. The conclusion that “apicoplast Fe-S dependent activities are specifically affected” by TgSufS knockdown (lines 330-331) is therefore overstating the conclusions that can be drawn from the data. The authors should note these limitations in their data (and temper their conclusions), or otherwise show that protein levels of TgPDH-E2 are unchanged by TgSufS knockdown (e.g. through epitope tagging approaches).

We agree it would have been better to ensure by immunoblot that only lipoylation (for which we now provide quantitative data - Figure 7A), and not the overall level of TgPDH-E2, is affected upon TgSufS depletion.

As mentioned above, we failed to modify the cKD TgSufS-HA cell line to tag TgPDH-E2 and we did not manage to obtain a new cKD TgSufS that we could use for a new tagging strategy based on chloramphenicol selection. We also tried to obtain the only published antibody raised against the TgPDH-E2 ([10.1128/EC.00061-07](https://dx.doi.org/10.1128%2FEC.00061-07)), but it is unfortunately not available anymore in the lab of Wolfgang Bohne.

However, as evidenced by our data (Figure 7A) lipoylated TgPDH-E2 goes down after as early as 1 day of ATc treatment and remains low/undetectable throughout. On the other hand, our quantitative proteomics data, which was performed after 3 days of ATc incubation, show the same amount of unique peptides for TgPDH-E2 (32 ± 2,5 for the control and 32,25 ± 2,75 for the mutant, ~60% of sequence coverage). This strongly suggests TgPDH-E2 levels are unchanged by TgSufS knockdown and is now mentioned in the manuscript (l.340-343).

--------------------

<b>Part III – Minor Issues: Editorial and Data Presentation Modifications<*/b></br>Please use this section for editorial suggestions as well as relatively minor modifications of existing data that would enhance clarity.<br/><br/*>

Reviewer #1: 1.	Title: Considering that the manuscript addresses the role of apicoplast and mitochondrial Fe-S biogenesis pathways in Toxoplasma fitness and biology and not their contribution to iron-sulfur cluster synthesis per se, the title should be modified.

We understand this reviewer’s point. Our paper does not directly address iron-sulfur cluster synthesis from a biochemical point of view in the mutants we have generated, yet our proteomics data clearly shows known Fe-S proteins are affected, which goes a little beyond just assessing fitness and differentiation. To be more inclusive, we thus modified the title to “Differential contribution of two organelles of endosymbiotic origin to iron-sulfur cluster synthesis **and overall fitness** in Toxoplasma”.

2.	The introduction is too long. The second paragraph, in particular, should be shortened.

The second paragraph has been streamlined, as well as some other parts of the introduction as suggested.

3.	Fig. S1 and S3A: Sequence alignments show N-terminal and C-terminal extensions of T. gondii proteins in black background (otherwise indicating conserved amino acids). This representation should be corrected. TgSufS has a large N-terminal extension. How does this compare with the previously characterized Plasmodium falciparum homolog (Charan et al., doi:10.1128/AAC.02711-13)?

Representation has been corrected on the two supplemental figures.

As for the SufS N-terminal extension, the *T. gondii* homologue is indeed longer than the *Plasmodium* homologue. We sequenced the cDNA and although we found a difference over the predicted sequence in ToxoDB (there is an additional intron in the 3’ region), it does not concern the region coding for the N-terminal part. The predicted molecular mass of the unprocessed protein is 76.7 kDa, which is perfectly compatible with what we see by immunoblot. Hence, it is very likely this N-terminal extension exists.

4.	In Fig.2A, there is a large size difference (~20 kDa) between the putative precursor and mature forms. Is this the result of removal of an additional stretch of amino acids beyond the apicoplast-targeting element in TgSufS? The sizes of the bands should be interpreted.

Given that the mature TgSufS form seems to run at around 65 kDa on a gel, it seems a large part (at least the two third or so) of the N-terminal extension is cleaved. We tried several plastid targeting signal prediction softwares for TgIscU, TgSufS and TgSufC, as we mention in the paper “no consensus position of the exact cleavage sites could be determined” (l. 177-178). Results are summarised in the table below. It is known that in plants for instance transit peptides can be separated from N-terminal signal peptides by a pro-domain of varying length. This may affect prediction as algorithms that analyse the N-terminal amino acid composition assume that potential transit peptides start at the first residue after the signal peptide. Because of the uncertainty concerning the cleavage sites, we prefer to avoid speculating and provide information only on simple yet experimentally-validated evidence: 1) consistent localisation predictions that match our experimental data; 2) presence of immature and mature forms for each of the proteins as detected by immunoblot.

|  | TgIscU | | TgSufS | | TgSufE | |
| --- | --- | --- | --- | --- | --- | --- |
| Software | Prediction | Cleavage | Prediction | Cleavage | Prediction | Cleavage |
| Localizer 1.0 | Mito | 31 | Plastid | 78 | Plastid | 42 |
| iPSORT | Mito | * | Plastid | * | Plastid | * |
| MitoProt II | Mito | 67 | - | - | - | - |
| ChloroP 1.1 | - | - | Plastid | 25 | Plastid | 37 |
| DeepLoc 1.0 | Mito | n/a | Plastid | n/a | Plastid | n/a |
|  |  |  |  |  |  |  |
| * First 30 amino acids analysed only | | |  |  |  |  |

5.	Fig. S3. What is the predicted cleavage site of the apicoplast targeting element? Indicating it in Fig.S3A is required for confirming expected protein sizes in Fig. S3D.

Please see reply to the point raised above.

6.	Fig. 4E. The Y-axis label on the plot reads “lysis plaques”. Do these represent the total number of plaques per treatment or are they % of control? The number of plaques should be plotted as % of control so that the statement in line 225 that “only 28% ±2% of plaques were formed after ATc removal” in cKD TgIscU-HA parasites is reflected in the figure.

Fig. 4E has been replaced in the new version of the manuscript and no longer mentions plaque numbers but now focuses on plaque area for the reversibility of the phenotype, including a longer washout period (7 days instead of 4), and a control with continuous presence of ATc.

7.	Line 68-69: “….for causing serious disease….in animals including humans.”

Corrected.

8.	Line 121: “…also present in the mammalian hosts...”

Corrected.

9.	Line 130: “…to parasite development and fitness…”

Corrected.

10.	Title: lines 133-134. In this section, functional homology is verified using E. coli mutants and not organellar counterparts...the title does not reflect that.

Agreed. Title has been changed to: “TgSufS and TgIscU are functional homologs of components of the iron-sulfur cluster synthesis pathways”. Figure 1 title has been modified too.

11.	Line 178: Similarly to or similar to?

Changed to “Similar to”

12.	Line 224 should be”…wells where the same initial dose…”

Phase has been removed.

13.	Line 388: “…were predicted to be cytoplasmic or nuclear.”

Corrected.

14.	Line 389-390:”… the cytosolic CIA Fe-S cluster assembly pathway is supposedly dependent from the SUF pathway [29]”. This is incorrect. Thus far, reports (including the reference cited) show that the CIA Fe-S assembly pathway is dependent on mitochondrial ISC, not SUF.

Absolutely. This has been corrected.

Also in Line 513-514: “In other eukaryotes, the SUF pathway provides a yet

unknown precursor molecule as a sulfur provider for the cytosolic CIA Fe-S cluster assembly pathway”. Mitochondrial ISC, not plastidic SUF, is the sulfur provider for cytosolic CIA.

Corrected too. Thanks again for pointing this out.

15.	Line 494: “…metabolic changes encountered by SUF-deficient parasites do not

cause their rapid demise..”. Shouldn’t this be ISC-deficient?

True. This has been corrected.

16.	Line 545: “..mitochondrial SUF”….should be mitochondrial ISC.

Corrected.

17.	Lines 545-547: RNA-interacting/regulating proteins (transcriptional regulators) and two putative ribosome biogenesis proteins are also less abundant in cKD TgSufS parasites. The cell stress response through post-transcriptional regulation thus may not be a mitochondrion-specific effect.

There are 16 versus 4 less abundant RNA-interacting/regulating proteins in the TgIscU mutant compared with the TgSufS mutant. Noticeably, many of these are related to stress granules, which is not the case for the TgSufS mutant. As this particular mutant commits more durably to stage conversion than the TgSufS mutant, like this reviewer suggests, it more likely reflects a general stress response which could play a part in stage conversion (and thus may also be induced by other bradyzoite-inducing stresses or factors). We have replaced “the mitochondrial ISC pathway mutant” by “the spontaneously-differentiating TgIscU mutant”, to avoid any confusion.

18.	Line 555-557: “This question is key to the pathology caused by T. gondii, as bradyzoites act as reservoirs susceptible to reactivate as and cause acute symptoms..”. The sentence should be rephrased.

This has been rephrased to: “This question is key to the pathology, because long term persistence of bradyzoites and their resistance to current treatments makes them a durable threat for their human hosts.”

Reviewer #2: A minor point to note is that there are quite a few grammar mistakes and typos – e.g. lines: 239, 305, 388.

We corrected these mistakes and others as we carefully went through the revised version of the manuscript.

Delayed dead or different point in the pathway

Sorry but this particular point is not clear.

The “washout” experiment is hard to interpret, as little is understood about ATc uptake and half-life. A suggestion here is that this caveated should be mentioned.

We included additional controls to the washout experiment (now presented in a new Figure 4E), including 14 days of continuous ATc incubation (medium was changed after 7 days and fresh ATc added). In these conditions no plaque was observed contrarily to the washout condition, indicating that ATc (or at least its effects) is likely stable for a long time.

The impact on apicoplast PDH-E2 lipoylation provided nice support to the proposed narrative. Still the interpretation in line 330-331 are extrapolation and should be revised.

Thank you. Our conclusion has been toned down: “This finding **suggests** apicoplast Fe-S-dependent activities **may be** specifically affected in our mutant before observing the general demise and loss of the organelle”.

Line 509 in the discussion refer to data that is not presented. PLOS pathogens typically discourages discussion of data that is not shown.

This was not essential to the discussion and has been removed.

Nomenclature: the authors primarily seem to be using names from plants e.g. ATM3, AE7, INDH, GRXS15, but in some places deviate from this (ISCU instead of ISU1 and ISCS instead of NFS1, NFU1 instead of NFU1 is chloroplastic). Additionally, here there is a different name for ISCS than a recent publication (Aw et al 2020) which called it NFS1 (while here call NFS1 in table S1 but ISCS in figure 1). Overall, a consistent naming system, that is also consistent with previously published Toxoplasma names is needed.

We agree with the reviewer. We decided to homogenize the nomenclature and to stick to the plant-related one (which makes sense as part of it is hosted in a plastid).

Sulphur line 55. Elsewhere is sulfur.

Changed to “sulfur”.

Figure 5 A) and C)- would aide reader comprehension to label which lines (IscU or SufS) are being imaged.

Done.

Line 108 “higher plants”- these days we are encouraged not to use terms like “higher” and “lower” referring to groups of organisms as it implies directional evolution and that one group is better than another.

We changed it to “land plants”.

Some references are incorrect- e.g. ref 62. This may have been a preprint when this manuscript was written, but was never published in “microbiology”- was published in Plos Pathogens. Same for ref 63 was never published in “cell biology”.

These are not journal names but BiorXiv sections. Now that these papers have been published, these references have been updated.

Table S1- ATM3 is not a cysteine desulfurase as stated.

True. This has been corrected.

Names in table S1 do not match figure 1- e.g. NFU4 vs NFU1

Sorry about that, NFU4 is the one in the ISC pathway. We corrected Figure 1. We also updated the scheme with the putative cytosolic localisation of TgHCF101, as suggested by Pyrih et al. ([10.1186/s12862-021-01777-x](https://doi.org/10.1186/s12862-021-01777-x)) and our own unpublished data.

Figure 1A- a nicely done illustration of pathway- given the unique shape of the mitochondrion in Toxoplasma (which is shown by IFA later in the paper) may be good to alter to show a lasso shaped mitochondrion.

Thanks. Done.

Table S6 – more informative primer names would be useful.

This is our internal lab nomenclature; this way they can easily be traced if anyone asks us for reagents. Some of the primers have been used both for constructs and diagnostic PCR, which does not allow assigning a simple function to each name. Whatever the primer names, all are referred to in the corresponding ‘materials and methods’ section for what they have been used, so identifying them should be quite straightforward.

Reviewer #3: 5. Lines 17-18. “Phenotypic analysis and quantitative proteomics allowed us to highlight striking differences in these mutants. Both Fe-S cluster synthesis pathways are necessary for optimal parasite growth in vitro, but their disruption leads to markedly different fates”. Striking probably isn’t the correct term here. Given that the plastid and mitochondrial pathways contribute differently to the biology of other organisms in which both pathways occur (e.g. plants), this observation doesn’t seem particularly “striking” or surprising.

This is a fair point, although ‘striking’ wasn’t meant here to suggest a particularly surprising result, but rather a ‘notable’ difference in the phenotype (which, we agree, may have been somewhat expected given the different functions of the organelles). We thus replaced it by ‘notable’ in the revised version.

6. Line 69: should be “responsible for causing serious disease”.

Corrected.

7. Line 74. “…that already contained a plastid …”

Text removed in the revised version.

8. Line 84. “the parasite’s metabolic needs” (apostrophe) or “the metabolic needs of the parasite”

Corrected.

9. Line 86. “which generates a proton gradient” perhaps? As written is sounds like the TCA cycle generates the gradient

Corrected.

10. Line 151. Given that the genome-wide screening approach does not provide direct or independent verification of the effects of gene loss on parasite fitness, perhaps “most components of the three pathways are [predicted to be] important for parasite fitness”

Good point. Corrected.

11. Line 152. Why are ‘apoproteins’ essential for optimal growth? Presumably the addition of FeS clusters to apoproteins to form the holoprotein is what enables these proteins to carry out their function? Perhaps “… several FeS proteins ... are essential …”

Absolutely. Corrected.

12. Line 154: The first sentence of this paragraph leads the reader to expect a description of mutants in T. gondii, but then goes on to explain a bacterial complementation experiment in E. coli. Perhaps a better leading sentence could be used?

This has been modified.

13. Line 155-156. “we targeted the SufS and IscU homologs” – unclear what is meant by ‘targeted’. “Investigated” would be a better term. Perhaps a brief description of the roles of SufS and IscU in the SUF and ISC pathways would help non-expert readers appreciate why these proteins are presumably essential to their respective pathways (and enable a better understanding of Figure 1A).

We now provide more background so that the reader would better understand the functions of these proteins (l.143-151).

14. Line 157. “We first sought to verify [that]] …” or “We first sought to [determine whether] …”

This has been changed.

15. Line 158-160. A brief description of the bacterial knockouts is required for readers to appreciate these data. e.g. describe that bacterial KOs of SufS and IscU are impaired in growth before stating what expression of the T. gondii homologues (partially?) restores growth. Also describe why the chelator is being added.

More background has been provided on the bacterial mutants in the revised version (l.154-158).

16. Figure S2A and elsewhere(S3B, S4A, S5A). Perhaps the diagrammatic representations of the genetic modifications could include the expected sizes of the PCR products so that the meaning of the diagnostic PCRs can be properly interpreted by the reader? i.e. what are the expected sizes of the PCR products in the modified loci?

Done.

17. Line 193: delete “conditional” here since this is mentioned earlier in the same sentence.

Corrected.

18. Line 213. “all cell lines were preincubated in ATc for 48 hours and released mechanically, before infecting new host cells and growing them for an additional 24 hours in ATc prior to parasite counting.” The description here is confusing. The figure and figure legend seem to imply that there was also a minus ATc condition. If so, perhaps “…preincubated in the absence or presence of ATc for 48 hr …”

Corrected.

19. Line 223-224. “comparing plaque number between the 7-day and 4-day washout conditions in wells …” This is confusing. Was there also a condition where ATc was washed out and parasites allowed to grow for 7 days? If so, this isn’t apparent in the figure. Perhaps the authors mean the 7 day incubation on ATc vs 7 day incubation plus 4 day washout? If so, this comparison also doesn’t seem to be represented in the figure (but probably should; see major comment 2 – determining plaque number between washed out and non-washed out experiments would be a valuable control).

New washout experiments were performed taking into account this reviewer’s comments (major comment #2) on additional controls to be included, but we decided to focus on plaque area this time (plaque numbers were found to be quite variable from one experiment to the other). The corresponding text was modified accordingly.

20. Figure 4E. It is not clear what ‘lysis plaques’ means on the y axis of the accompanying graph. Are the authors referring to plaque number? If so, in what context? In wells of a plate? As noted in major comment 2, a better way of expressing this would be compared to a relevant control (e.g. where parasites were incubated in the absence of ATc, but otherwise treated the same, or where there was no washout of ATc).

Line 224. “where” not “were”?

This has been removed from the revised version.

21. Figure 5A, C. Given that this figure depicts data from both the TgSufS and TgIscU mutants, it might help to label the IFAs in the figure to indicate which mutant is being examined?

Done.

22. Line 239. “defect” rather than “default”?

Changed.

23. Line 242-245. In our experience, spontaneous stage conversion to bradyzoites happens very rarely in the RH TATiΔku80 strain. On first read, this seemed like a surprising observation, and the interpretation that these could be tissue cysts seemed speculative. Given that the authors revisit this later in the manuscript, perhaps they could flag here that they will return to this possibility later?

Thanks for suggesting this. Done.

24. Lines 294-298 – the authors comment that SRS19F expression decreased the most, yet this protein is supposed to be expressed in bradyzoites. So wouldn’t the opposite result be expected if the parasites are transitioning to bradyzoites?

Very little is known about this particular SRS. Analysis of VEG strain oocyst ESTs has previously identified SRS19 as sporozoite-specific ([10.1101/gr.693203](https://dx.doi.org/10.1101%2Fgr.693203)), and ToxoDB transcriptomic data suggests it may be predominantly expressed in some sexual stages.

It is not specific to bradyzoite and we realize mentioning bradyzoites was confusing, we thus removed it.

25. Line 305. Should read “but several [are] stage-specific”

Corrected.

26. Lines 332-345 and line 346-359. These paragraphs speculate on the biological implications of SufS knockdown, and belong in the discussion. As mentioned in major comment 3, the discussion here would be strengthened if these ideas were investigated experimentally (e.g. are the GPI-anchored/glycosylated rhoptry proteins or ER-localised lipid-related enzymes discussed really depleted upon TgSufS knockdown?).

These paragraphs have been streamlined and a large part was transferred to the discussion as suggested. As mentioned above, we did try to edit genetically the original TgSufS knock-down cell line to tag specific targets, but due to the lack of availability of efficient selection markers in that cell line, it did not work. Unfortunately, we also failed to generate a new and untagged TgSufS mutant cell line in which it would be easier to tag new candidates.

27. Line 357. “in compensation from” should read “in compensation for” or “compensating for”

Corrected.

28. Line 368. “This ETC comprises five complexes” – apicomplexans lack Complex I.

Absolutely, we rewrote this phrase to make less confusing: “This ETC comprises four complexes in apicomplexa (which typically lack complex I)”

29. Figure 9. The JC-1 assay can also be used as a marker for cell viability (e.g. if broader cellular metabolism is impaired, mitochondrial membrane potential will decrease). Can the authors rule out the possibility that the decrease in mitochondrial membrane potential results from general cell ‘death’ rather than specifically from depletion of ETC proteins? In the absence of more direct data, the authors should be more circumspect here. They might also consider the recent Aw et al study (PMID 33222310), which found that depletion of another ISC pathway protein led to specific depletion of the FeS protein of Complex II, and a specific impairment of mitochondrial O2 consumption in T. gondii (which would support the hypothesis they are proposing here).

The JC-1 assay clearly shows the mitochondrial membrane potential is affected, but it is true we cannot completely rule out that some of this could be due to some cell death. During our cytometry analysis, the light scatter profiles between the +/- ATc populations were similar, which would indicate there was no extensive cell death (granulometry often changes significantly with dead parasites), but this is of course not completely reliable.

Also, given what we know of this mutant, including the replication and morphological studies we have performed, a sharp decrease in viability after only two days of ATc treatment would be surprising, although it might happen in the long term. On the contrary JC-1 labelling changes already reach a plateau after two days of ATc treatment. Yet again this is no absolute proof.

Of note, we also tried to perform SeaHorse experiments and could see an encouraging trend with a decrease in mitochondrial respiration and an increase in extracellular acidification rate (suggesting an increased glycolytic activity) with the IscU mutant. However, due to recurrent parasite quantification and normalization problems, these results are not of a good enough quality to be included in the present publication.

That being said, Aw et al. have very nicely demonstrated that depletion of another ISC pathway protein led to specific depletion of TgSDHB, and clearly demonstrated the impact on mitochondrial respiration by SeaHorse experiments, so the reviewer is right we can mention this further to support our own findings. The text has been modified accordingly (l.392-397).

30. Line 355. delete “these”.

Done.

31. Line 370. It is unclear why cytochrome b and c1 are highlighted here. Complex III is composed of other subunits as well (as noted later in the paragraph). Perhaps just “part of Complex III”, or “part of the catalytic machinery of Complex III, along with cytochromes b and c1”.

Changed.

32. Line 389. “the cytosolic CIA Fe-S cluster assembly pathway is supposedly dependent from the SUF pathway”. The meaning here isn’t clear. “Independent from” perhaps? Or do the authors mean to refer to the ISC pathway? Regardless, this is all rather speculative – many cytosolic and nuclear proteins do not require FeS clusters. This probably belongs in the discussion.

It was a mistake, we meant ‘**ISC’** pathway, and this has been corrected. Of course many cytosolic and nuclear are not Fe-S proteins, but some may be indirectly affected. We agree this is speculative, but as some putative nuclear Fe-S enzymes (like a DNA glycosylase/endonuclease) seem to have a lower expression upon TgISCU deletion, so we think it is worth briefly mentioning it in the ‘results’ section.

33. Line 393-4. Should read “The expression of several bradyzoite-specific [proteins]…”

Corrected.

34. Figure 10. The data here are really exciting. The authors use nitric oxide treatment to induce bradyzoite formation, and note that the phenotypic similarity between NO treatment and IscU knockdown could be explained by the effects of NO on FeS cluster proteins (Lines 402-402 and Figure 10B). The authors note in their discussion that inhibitors of the mitochondrion induce bradyzoite differentiation (Lines 442-443). These include inhibitors of the ETC (e.g. atovaquone). Is it possible that the differentiation that the authors see upon IscU knockdown results from impairment of the ETC? This is something the authors could explore more extensively in their discussion.

Thanks for your positive comments. In the revised version of the paper we are now including the analysis of other mitochondrial mutants with regards to stage conversion. Interestingly, the TgQCR11 mutant (affected in the complex III of the mitochondrial respiratory chain) is very similar to the TgISCU mutant in the kinetics for appearance of early markers of differentiation. We also included atovaquone as a control for early differentiation, but parasites could not be kept long enough in culture for assessing late bradyzoite markers (even in the presence of atovaquone, tachyzoites remained and caused extensive host lysis). This brings further evidence that altering ETC function may trigger stage conversion and this explored further in the discussion (l. 626-637).

35. Lines 470-475. “Our quantitative proteomic analysis shows potential compensatory mechanisms may be used by the parasites in response this early perturbation of the apicoplast lipid metabolism that precedes organelle loss … upon depletion of TgSufS we observed a pattern of overexpression for ER-located enzymes involved in the synthesis of several phospholipids and ceramides” The authors could dig a little deeper here. Which proteins in particular are overexpressed? Does this point to particular lipid classes as being generated by the parasite ER upon the presumed loss of the apicoplast pathway?

If fatty acid (FA) synthesis in the apicoplast is impacted, one can expect a decrease in short chain FAs, and a compensatory uptake of precursors from the host cell for further elongation in the endoplasmic reticulum. This is something that we are currently exploring further (performing lipidomic analysis of Suf mutants), but deserves a full project in its own right. This is mentioned in the discussion (l.545-559).

36. Line 494-495. “The metabolic changes encountered by SUF-deficient parasites do not cause their rapid demise, as they are able to initiate conversion to the bradyzoite stage”. What is the evidence for this? That some bradyzoite genes are upregulated upon SufS knockdown? The data in Figure 10B suggest that bradyzoite differential does not occur upon SufS knockdown. Without more direct evidence that bradyzoite initiation occurs upon SUF knockdown, the authors should temper their conclusions (or perhaps remove this section, since these points are revisited later in the discussion (from Line 523).

Apologies, we meant **ISC-deficient**, not **SUF-deficient**. This has been corrected.

37. Line 496. Delete “anyway” from the end of the sentence.

Done.

38. Line 504. Perhaps “One possible reason”?

Changed.

39. Lines 513-514. “the SUF pathway provides a yet unknown precursor molecule as a sulfur provider for the cytosolic CIA Fe-S cluster assembly pathway”. The ISC pathway?

Yes, sorry. This mistake has been corrected.

40. Line 556. Delete ‘as’.

This part has been rephrased.

41. Line 669. The authors could provide details of how the washout plaque assay experiment was performed in this section.

Details have been added (l.790-794).
